# Analysis of the Relationships between DNA Double-Strand Breaks, Synaptonemal Complex and Crossovers Using the *Atfas1-4* Mutant

**DOI:** 10.1371/journal.pgen.1005301

**Published:** 2015-07-06

**Authors:** Javier Varas, Eugenio Sánchez-Morán, Gregory P. Copenhaver, Juan L. Santos, Mónica Pradillo

**Affiliations:** 1 Departamento de Genética, Facultad de Biología, Universidad Complutense de Madrid, Madrid, Spain; 2 School of Biosciences, University of Birmingham, Birmingham, United Kingdom; 3 Department of Biology and the Carolina Center for Genome Sciences, University of North Carolina at Chapel Hill, Chapel Hill, North Carolina, United States of America; 4 Lineberger Comprehensive Cancer Center, University of North Carolina School of Medicine, Chapel Hill, North Carolina, United States of America; Stanford University School of Medicine, UNITED STATES

## Abstract

Chromatin Assembly Factor 1 (CAF-1) is a histone chaperone that assembles acetylated histones H3/H4 onto newly synthesized DNA, allowing the *de novo* assembly of nucleosomes during replication. CAF-1 is an evolutionary conserved heterotrimeric protein complex. In Arabidopsis, the three CAF-1 subunits are encoded by *FAS1*, *FAS2* and *MSI1*. *Atfas1-4* mutants have reduced fertility due to a decrease in the number of cells that enter meiosis. Interestingly, the number of DNA double-strand breaks (DSBs), measured by scoring the presence of γH2AX, AtRAD51 and AtDMC1 foci, is higher than in wild-type (WT) plants, and meiotic recombination genes such *AtCOM1/SAE2*, *AtBRCA1*, *AtRAD51* and *AtDMC1* are overexpressed. An increase in DSBs in this mutant does not have a significant effect in the mean chiasma frequency at metaphase I, nor a different number of AtMLH1 nor AtMUS81 foci per cell compared to WT at pachytene. Nevertheless, this mutant does show a higher gene conversion (GC) frequency. To examine how an increase in DSBs influences meiotic recombination and synaptonemal complex (SC) formation, we analyzed double mutants defective for AtFAS1 and different homologous recombination (HR) proteins. Most showed significant increases in both the mean number of synapsis initiation points (SIPs) and the total length of AtZYP1 stretches in comparison with the corresponding single mutants. These experiments also provide new insight into the relationships between the recombinases in Arabidopsis, suggesting a prominent role for AtDMC1 *versus* AtRAD51 in establishing interhomolog interactions. In Arabidopsis an increase in the number of DSBs does not translate to an increase in the number of crossovers (COs) but instead in a higher GC frequency. We discuss different mechanisms to explain these results including the possible existence of CO homeostasis in plants.

## Author Summary

Meiosis is a special cell division common in all sexually reproducing organisms. It consists of two successive rounds of chromosome segregation, preceded by a single DNA replication event. Homologous recombination is a key process that occurs during the first meiotic division. It guarantees the association of the homologous chromosomes by chiasmata, the cytological manifestations of reciprocal interchanges (crossovers, COs). The formation of COs during meiosis is fine-tuned by several mechanisms. One of them, reported in some model organisms, is CO homeostasis, which ensures a consistent number of COs despite variability in early recombination events. Here we described the analysis of *Atfas1-4*, a mutant defective for the histone chaperone CAF-1 (Chromatin Assembly Factor 1), which has an increase in double-strand breaks (DSBs), without a corresponding increase in COs. Interestingly, *Atfas1-4* has a higher gene conversion (GC) frequency. These results demonstrate that Arabidopsis meiocytes are able to maintain WT levels of COs even when DSBs numbers are increased. Furthermore, we provide evidence for a prominent role for AtDMC1 in establishing interhomolog interactions in Arabidopsis.

## Introduction

Histone chaperones are a family of proteins that facilitate appropriate interactions between histones and DNA by regulating the assembly and disassembly of chromatin in response to cellular requirements [1–3]. CAF-1 is a heterotrimeric histone chaperone complex that mediates nucleosome assembly on newly replicated DNA in fungi, animals and plants [4].

The CAF-1 complex is composed of: FASCIATA 1 (AtFAS1), AtFAS2, and the MULTICOPY SUPPRESOR OF IRA1 (AtMSI1) [5]. The large subunit AtFAS1 binds acetylated histones H3/H4 and interacts with Proliferating Cell Antigen (PCNA) [6, 7]. The AtFAS2 subunit enables protein-protein interactions within CAF-1 and with Anti-Silencing Function 1 (ASF1), another major evolutionarily conserved H3/H4 histone chaperone, which also participates in replication-independent nucleosome assembly [8–11]. MSI1, the small subunit of CAF-1, forms part of other complexes involved in chromatin dynamics [12]. Mutants deficient in AtFAS1 and AtFAS2 show a fasciated phenotype characterized by shoot apical meristem defects including altered phyllotaxy, stem broadening and bifurcation, and alterations in floral organ numbers [13]. In addition, CAF-1 components also control genome replication at multiple developmental steps [14, 15], and maintain the correct pattern of heterochromatin silencing [16]. For instance, loss of AtFAS1 leads to a switch to the endocycle program [17]. Genomic DNA from these mutants is hypersensitive to DNA digestion, suggesting a less compact chromatin conformation [16, 18]. Moreover, *Atfas1* and *Atfas2* are hypersentive to genotoxic agents and DSB-inducing treatments [4, 18, 19]. Depletion of either subunit increased the frequency of somatic homologous recombination (HR) ~40-fold, as well as increased T-DNA integration [19]. These findings suggest that the loss of CAF-1 activity produces defects in chromatin assembly that lead to genomic instability. *Atfas1-4* appears to be the strongest allele since it exhibits a severe developmental phenotype and ~96-fold higher intrachromosomal HR [5, 18]. Since several HR genes display normal expression in the *Atfas1-4* mutant, it has been suggested that chromatin conformation is a key factor limiting HR in plants [18]. By contrast, single mutants in the ASF1 subunits AtASF1a or AtASF1b exhibit no apparent somatic abnormalities. However, double mutants show reduced growth and defects in organ development, and overexpression of genes involved in S-phase checkpoints and DNA repair by HR [11].

Additionally, CAF-1 deficiency leads to cell cycle arrest during post-meiotic pollen development resulting in formation of only one sperm cell [20]. Here, we use the *Atfas1-4* mutant to investigate the influence of an increase in DSB frequency on meiotic phenotypes in Arabidopsis. The characterization of double mutants defective for both AtFAS1 and either AtRAD51 or AtDMC1 allowed us to infer new insights about the interplay between the action of recombinases and the regulation of synapsis. Furthermore, the analysis of *Atfas1-4*, with a normal chiasma frequency, and a significant increase in the frequency of gene conversion (GC) events, suggests the possible existence of a homeostatic control of crossovers (COs) in Arabidopsis.

## Results

### Fertility phenotype of *Atfas1-4* plants

*Atfas1-4* plants have significantly fewer ovules per gynecium (31.58 ± 2.76 *vs*. 50.75 ± 2.04; W = -72.02; P < 10^−4^), and pollen grains per anther (14.17 ± 1.69 *vs*. 43.3 ± 1.95; W = 12.5; P = 3.9 x 10^−3^) compared to wild-type (WT) plants (S1 Fig). Likewise, the mean silique length (cm) and number of seeds are also significantly diminished in *Atfas1-4* compared to WT (0.71 ± 0.02 *vs* 1.30 ± 0.02; t = 18.804; P < 10^−4^; and 11.58 ± 0.4 *vs*. 48.8 ± 1.59; t = 22.046; P < 10^−4^, respectively). Consistent with previous phenotypic analysis of *Atfas1* plants, we observed that the flowers had only five anthers instead of the six consistently observed in WT (see Figure S1A in [4]). These results indicate that *Atfas1-4* plants have reduced fertility.

### Meiosis in pollen mother cells (PMCs)

**Fig 1:**
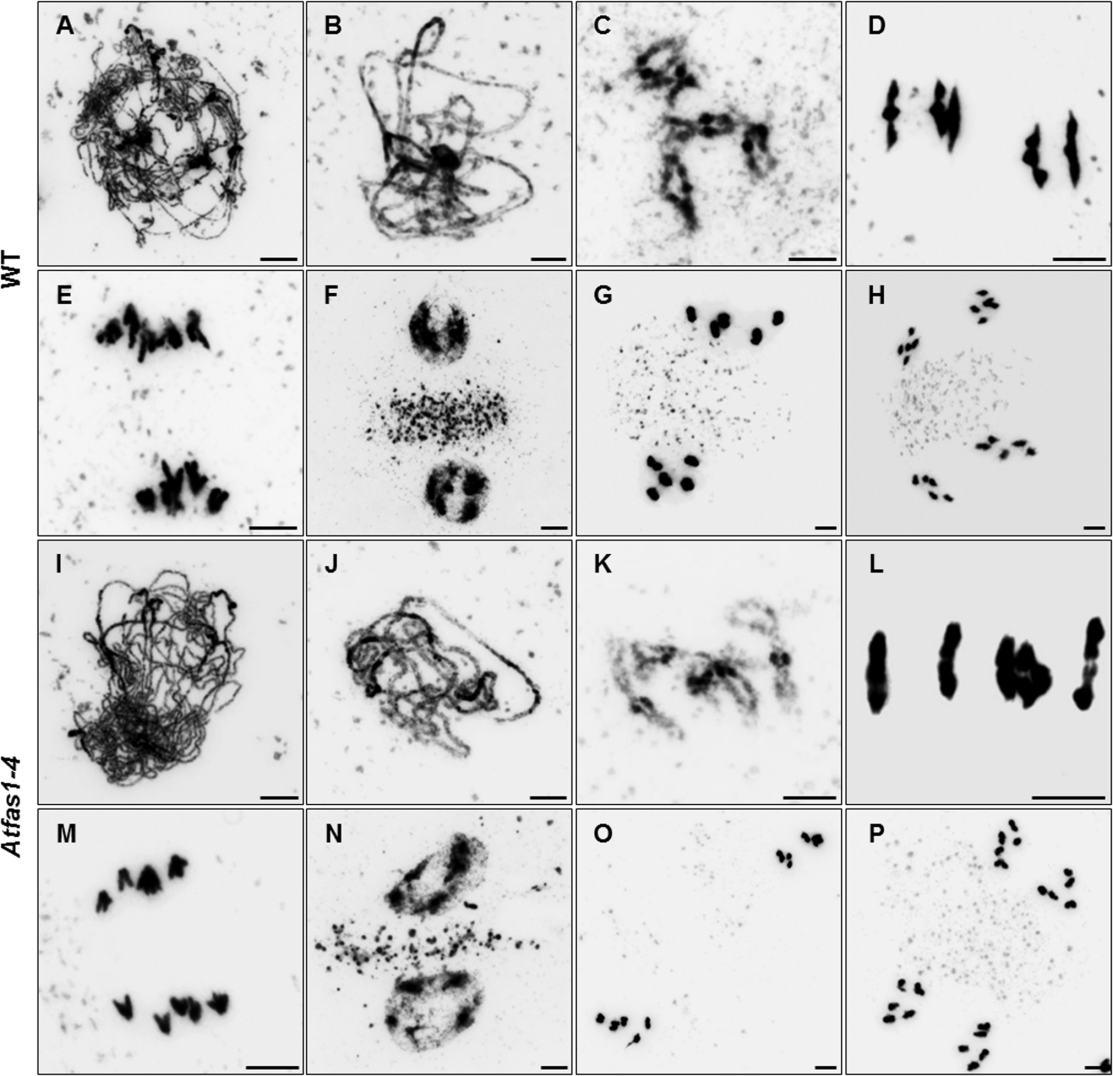
*Atfas1-4* does not show cytological meiotic alterations. (A-H) Chromosome spread preparations from WT and (I-P) *Atfas1-4* PMCs. (A, I) Leptotene. (B, J) Pachytene. (C, K) Diplotene. (D, L) Metaphase I. Five ring bivalents in WT and four ring bivalents in *Atfas1-4*. (E, M) Anaphase I. (F, N) Prophase II. (G, O) Metaphase II. (H, P) Anaphase II. Bars = 5 µm.

To assess the ability of *Atfas1-4* plants to progress through meiosis we compared 4’6-diamidino-2-phenylindole (DAPI) stained chromosome spreads of PMCs from WT and mutant plants. The cytological analysis revealed that in *Atfas1-4* chromosome pairing, synapsis and recombination between homologs occurred normally, as did the first and second divisions and tetrad formation (n > 400; Fig 1). Normal meioses were also observed in *Atfas2-1*, and in a line in which *AtASF1a* and *AtASF1-b* were inactivated by RNAi (S2 Fig). Therefore, the fertility decrease exhibited by these mutants is not due to a defect in meiosis.

Since *Atfas1-4* displays a more “open chromatin” configuration in somatic cells [19], we looked for changes in the chromatin of meiotic cells, particularly during pachytene, a stage when bivalents are individualized and decondensed. We hybridized chromosome spreads with the centromeric probe pAL1 (180 bp) and observed fading of the chromomeric pattern. The centromeric fluorescence in situ hybridization (FISH) signals are weaker, less defined, and more extended in *Atfas1-4* than in WT (1.26 ± 0.59 µm *vs*. 0.854 ± 0.059 µm; t = -2.126; P = 0.040; n = 30; S3 Fig). These observations suggest a gross change in chromosome condensation states during pachytene.

### Expression analysis of meiotic genes involved in HR

**Fig 2:**
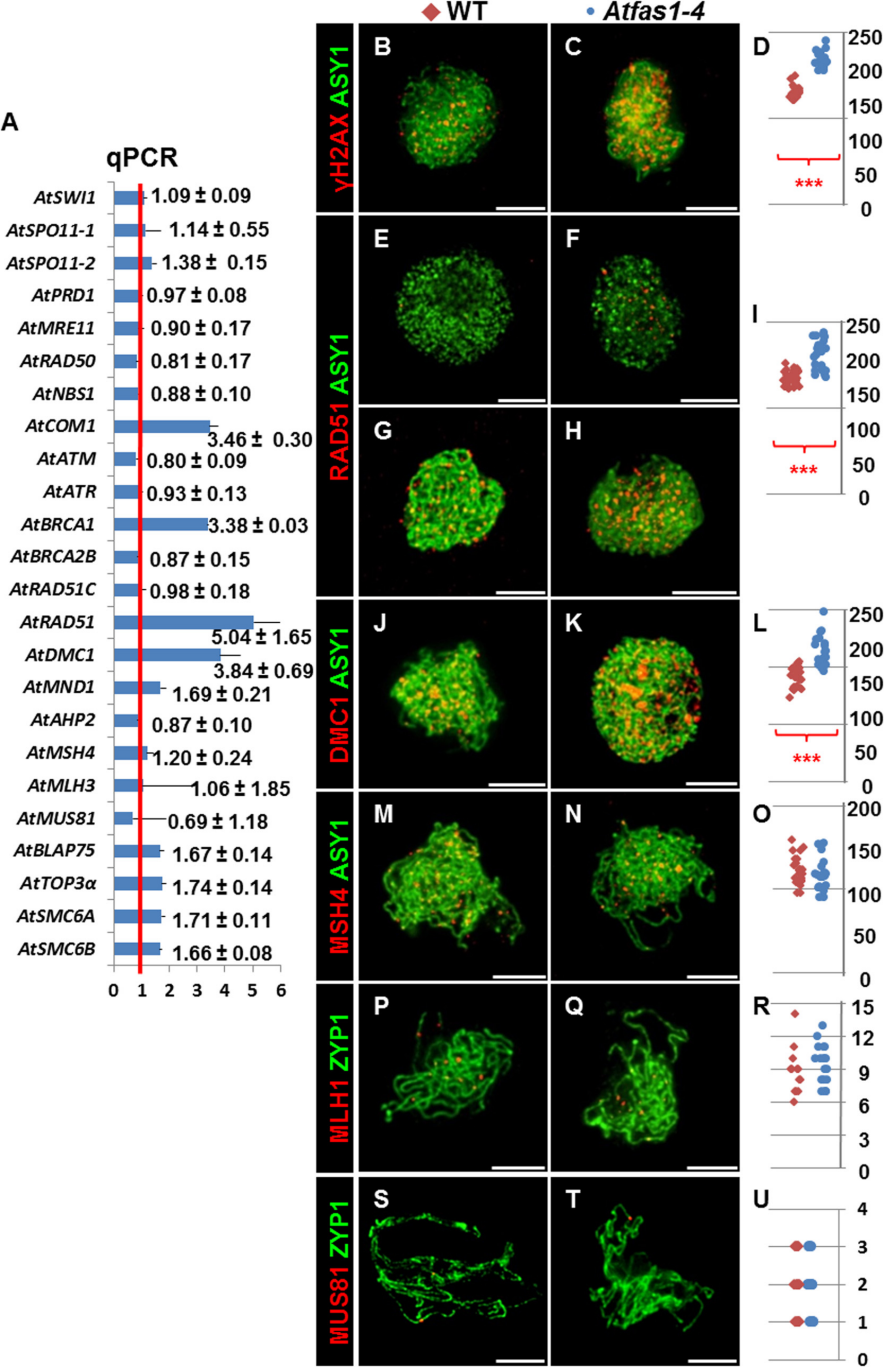
Gene expression and protein quantification for several genes involved in HR in *Atfas1-4*. (A) Expression analysis of genes encoding meiotic proteins in WT and *Atfas1-4* bud samples. Values are the average of assays carried out in triplicate using five different cDNA preparations. The red line is the reference for the fold change respect to the WT after normalization to 18S rRNA. The means corresponding to changes in gene expression and their standard errors are indicated. (B-Q) Dual immunolocalization of AtASY1 and AtZYP1 with HR proteins in WT and *Atfas1-4* prophase I nuclei. (B, C) AtASY1 (green) and γH2AX (red) on WT and *Atfas1-4* at leptotene. (E, G) AtASY1 (green) and AtRAD51 (red) on WT and (F, H) on *Atfas1-4* at G_2_ or leptotene, respectively. (J, K) AtASY1 (green) and AtDMC1 (red) on WT and *Atfas1-4* at leptotene. (M, N) AtASY1 (green) and AtMSH4 (red) on WT and *Atfas1-4* at late zygotene. (P, Q) AtZYP1 (green) and AtMLH1 (red) on WT and *Atfas1-4* at pachytene. (S, T) AtZYP1 (green) and AtMUS81 (red) on WT and *Atfas1-4* at pachytene. Bars = 5 µm. (D, I, L, O, R, U) Total foci per nucleus in WT (red) and *Atfas1-4* (blue) PMCs are indicated. Each dot is the count from a single nucleus. P values are from two-sided Wilcoxon Mann-Whitney tests (***P < 0.001).

Kirik and colleagues [18] reported that *Atfas1-4* displays one of the strongest intra-chromosomal HR phenotype of all chromatin mutants analyzed in plants to date. Therefore, we tested whether meiotic recombination is also enhanced. As a first approximation, we used real-time polymerase chain reaction (qPCR) to quantify the transcripts of several meiotic recombination genes in flower bud samples. After analyzing 15 measurements for each target gene from three experimental and five biological replicates, we detected a statistically significant overexpression of the meiosis-specific gene *AtDMC1* (3.842 ± 0.794), and also of DNA repair genes, including: *At*C*OM1* (3.460 ± 0.304), *AtBRCA1* (3.379 ± 0.025), *AtRAD51* (5.043 ± 0.656), *AtSMC6A* (1.719 ± 0.114), and *AtSMC6B* (1.664 ± 0.075) (numbers represent fold variation over WT and after normalization; Figs 2A and S4). The expression levels of three other genes: *AtMND1* (1.687 ± 0.206 *vs*. 1 ± 0.340), *AtBLAP75* (1.665 ± 0.138 *vs*.1 ± 0.404) and *AtTOP3α* (1.736 ± 0.141 *vs*. 1 ± 0.235) were suggestive of increases, but not statistically significant (see Materials and Methods for more details).

### Immunodetection of meiotic proteins in PMCs

To estimate the number of DSBs, we counted foci corresponding to phosphorylated histone H2AX (γH2AX), a sensitive marker that can be used to examine the DNA damage produced and the subsequent repair of the DNA lesion, and the recombinases AtRAD51 and AtDMC1 which are involved in DNA strand invasion during the beginning of the recombination process (S4 Fig). We also carried out immunolocalization of AtMSH4, which promotes the formation of Type I COs (CO^1^) that are sensitive to CO interference at zygotene, and AtMLH1 which marks CO^1^s at pachytene. To establish the chronology of prophase I we used AtASY1, a structural protein related to the axial/lateral element (AE/LE) of the synaptonemal complex (SC), and AtZYP1, which forms part of the central element (CE) of the SC, as cytological markers. We used *Atspo11-1-5* as a negative control, as DSB formation is blocked in this mutant (S5 Fig).

In both *Atfas1-4* and WT, AtASY1 adopted a linear configuration, and appeared in intimate association with the AEs of the SC at leptotene (Fig 2B–2N). However, the signal strength of γH2AX foci was more intense in the mutant (Fig 2B and 2C). As expected, no γH2AX signal was observed in *Atspo11-1-5* (n = 15; S5 Fig). Quantification of γH2AX foci suggests that there are significantly more DSBs in *Atfas1-4* compared to WT: 57.70% (W = 112.0; P = 1.42 x 10^−5^; Fig 2D and S1 Table).

AtRAD51 and AtDMC1 were detected in both *Atfas1-4* and WT at early prophase (Fig 2E–2K). Again as expected, AtRAD51 and AtDMC1 foci were absent in *Atspo11-1-5* (S5 Fig). Furthermore, in *Atfas1-4* abundant AtRAD51 signals appeared at G_2_, before leptotene (Fig 2F) whereas they were scarce in WT (Fig 2E). We also quantified the number of foci corresponding to both recombinases. In *Atfas1-4* the number of foci of AtRAD51 and AtDMC1 was significantly higher than in WT, 41.13% (W = 442.5; P = 3.98 x 10^−9^; Fig 2I), and 33.56% (W = 180.5; P = 2.24 x 10^−6^; Fig 2L), respectively (S1 Table).

We also carried out simultaneous detection of AtMSH4 and AtASY1, which showed diffuse and discontinuous signals in chromosome regions which had started to synapse (Fig 2M and 2N). AtMLH1 and AtMUS81 localizations were performed with AtZYP1, which appears as a linear signal at pachytene (Fig 2P, 2Q, 2S and 2T). No significant differences for AtMSH4 (W = -34.5; P = 0.31; Fig 2O), AtMLH1 (W = 13.5; P = 0.66; Fig 2R), or AtMUS81 were observed, (W = -9.0; P = 0.71; Fig 2U) (S1 Table).

### Chiasma analysis

**Fig 3:**
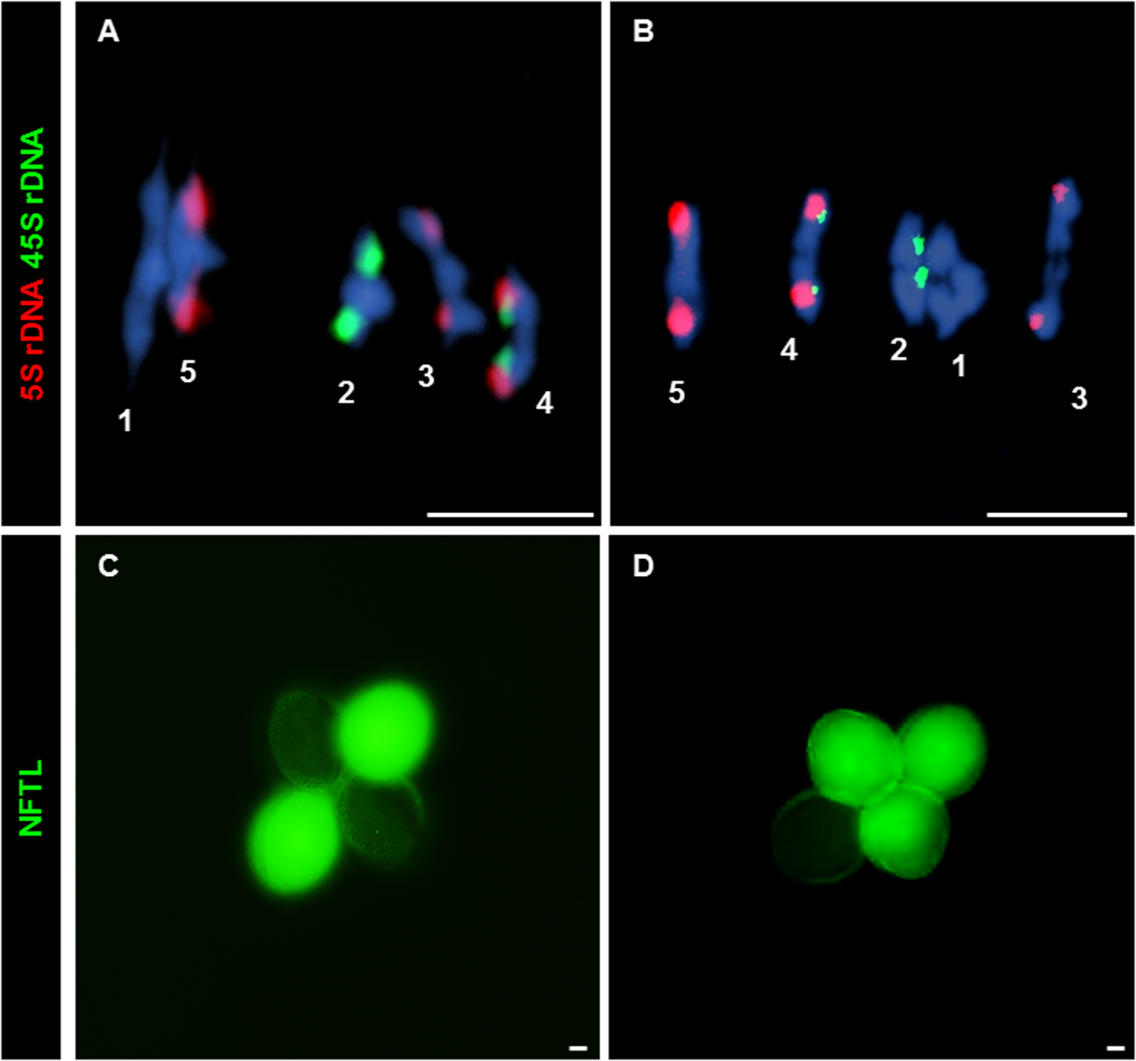
CO and NCO measurements: Chiasma and GC scoring by FISH and NFTLs. (A, B) FISH using 5S (red) and 45S (green) rDNA probes for chromosome identification and chiasma scoring. (A) WT metaphase I. (B) *Atfas1-4* metaphase I. Four ring bivalents (with at least one chiasma in both arms) and one rod bivalent (chromosome 3) are observed. (C, D) Two different examples for the GC test loci using NFTL. (C) Tetrad without GC (2:2). (D) Tetrad with GC (3:1). Bars = 5 µm.

We used FISH with probes for 45S and 5S rDNA to distinguish the chromosomes of *Arabidopsis*, and counted chiasmata to estimate the mean total COs per cell [21–23] (Fig 3A and 3B). Data were collected from three plants per genotype. Since there were not significant differences in the mean number of chiasmata (the cytological expression of COs) per cell between plants, data were grouped. The mean chiasma frequencies per cell, per bivalent and per bivalent arm are shown in Table 1. There were no significant differences between WT and *Atfas1-4* for any of these parameters. Furthermore, we did not detect changes in the distribution of COs over the chromosomes, which are located in either distal or subdistal regions. These results are consistent with the previous AtMLH1 and AtMUS81 immunolocalization data (Fig 2P–2U).

**Table 1:** Mean chiasma frequencies per cell, per bivalent, and per bivalent arm.

	Bivalent											
	1		2		3		4		5		Total	n^c^
	S^a^	L^b^	S	L	S	L	S	L	S	L		
	0.99	1.54	0.61	1.14	0.9	1.26	0.48	1.01	0.97	1.3		
WT	2.52		1.75		2.16		1.49		2.28		10.2 ± 0.4	69
	(0.25)		(0.17)		(0.21)		(0.15)		(0.22)			
	0.87	1.50	0.77	1.17	0.90	1.27	0.77	1.10	0.93	1.33		
*Atfas1-4*	2.37		1.93		2.17		1.87		2.27		10.6 ± 0.28	30
	(0.22)		(0.18)		(0.20)		(0.18)		(0.21)			

^a^ Short arm

^b^ Long arm

^c^ Number of cells analyzed

### Tetrad analysis of meiotic GC events

Because we observed results consistent with increased levels of DSBs but did not detect an increase in COs we tested whether GC frequencies change in *Atfas1-4* relative to WT. GCs can accompany either COs or non-crossovers (NCOs), but in the absence of an increase in COs an increase in GCs can be interpreted as an increase in NCO repair of DSBs. To detect GCs we used the *quartet1* non-fluorescent tagged lines (NFTLs) in a system described by Sun and colleagues [24]. Pollen tetrads from plants heterozygous for fluorescent and non-fluorescent alleles at a transgene locus will segregate fluorescence in a 2:2 ratio (Fig 3C). If GC occurs at the test locus, a non-Mendelian 3:1 ratio is observed (Fig 3D). The chromosomal localization of the different NFTL alleles is shown in S6 Fig. We analyzed five plants obtained from different crosses (S2 Table). We found no difference between plants so we grouped the data (Table 2).

**Table 2:** GC frequencies in *Atfas1-4*.

NFTL allele	Chr.	GC events	Tetrads scored	Raw frequencies (GC obs)	Adjusted frequencies (GC obs)^a^	Meioses^b^	Meioses (WT)^c^
NFTL 567	1	12	11781	1.02 x 10^−3^	2.04 x 10^−3^	490.88	4379
NFTL 3411	2	7	7846	8.92 x 10^−4^	1.78 x 10^−3^	560.43	2512
NFTL 424	4	6	7315	8.20 x 10^−4^	1.64 x 10^−3^	609.58	936

^a^ Adjusted frequencies after doubling the number of 3:1 tetrads to account for the elimination of 1:3 tetrads from the analysis. See [24] for more details.

^b^ Number of meioses required to observed one GC.

^c^ Data taken from [24].

Comparison of *Atfas1-4* data with previously published WT data revealed a 9-fold increase in GC frequency at the chromosome 1 test locus in *Atfas1-4* (χ^2^ = 49.43; P < 10^−4^). A test locus on chromosome 2 also showed a 4.5-fold increase (χ^2^ = 15.34; P = 10^−4^). A third test locus on chromosome 4 showed a slight (1.53-fold) but non-significant increase (χ^2^ = 1.04; P = 0.308). This result may be explained by the fact that the chromosome 4 locus has the highest GC frequency in WT. It should also be noted that GC frequencies in WT are not uniform between loci [24] (χ^2^ = 51.98; P < 10^−4^), but that such differences were not observed in *Atfas1-4* (χ^2^ = 0.21; P = 0.902).

### Meiotic analysis in PMCs of different double mutants

To determine if the additional DSBs observed in *Atfas1-4*, as measured by counting γH2AX foci, are dependent on AtSPO11-1 we obtained an *Atfas1-4 Atspo11-1-5* double mutant. We compared the phenotype of this double mutant to WT and to other *Atfas1-4* double mutants in genes involved in downstream steps in the meiotic recombination process, including *Atdmc1-2* (knockout allele, KO), *Atrad51-2* (knockdown allele, KD) and *Atrad51-3* (KO).

#### *Atfas1-4 Atspo11-1-5*

**Fig 4:**
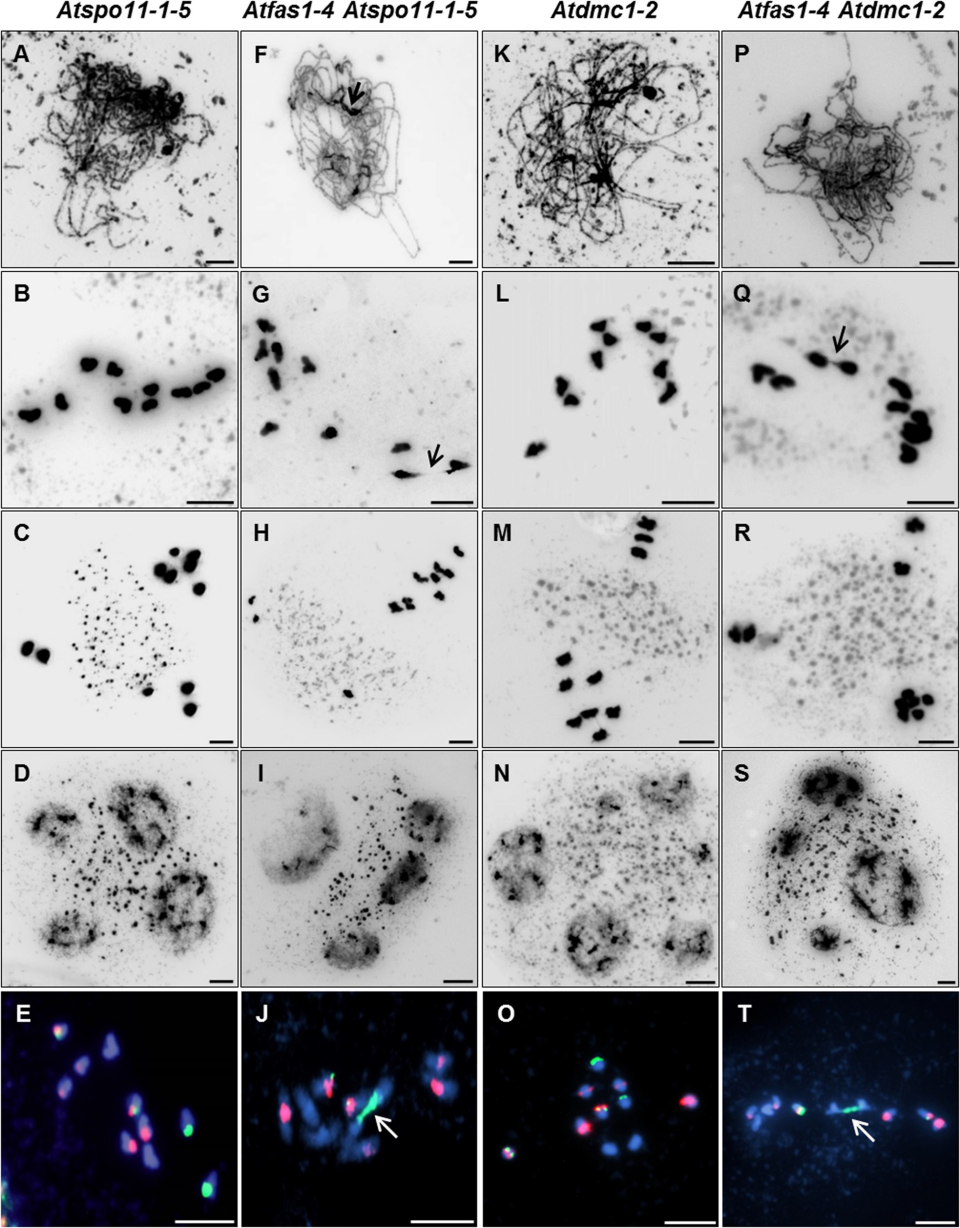
*Atfas1-4 Atspo11-1-5* and *Atfas1-4 Atdmc1-2* display CO on some occasions. Meiotic spreads of PMCs stained with DAPI. (A-D) *Atspo11-1-5*. (F-I) *Atfas1-4 Atspo11-1-5*. (K-L) *Atdmc1-2*. (P-S) *Atfas1-4 Atdmc1-2*. (E, J, O, T) FISH metaphases I using 5S (red) and 45S (green) rDNA probes for chromosome identification in these four backgrounds. Bivalents are observed in the two double mutants (arrows). Bars = 5 µm.

**Fig 5:**
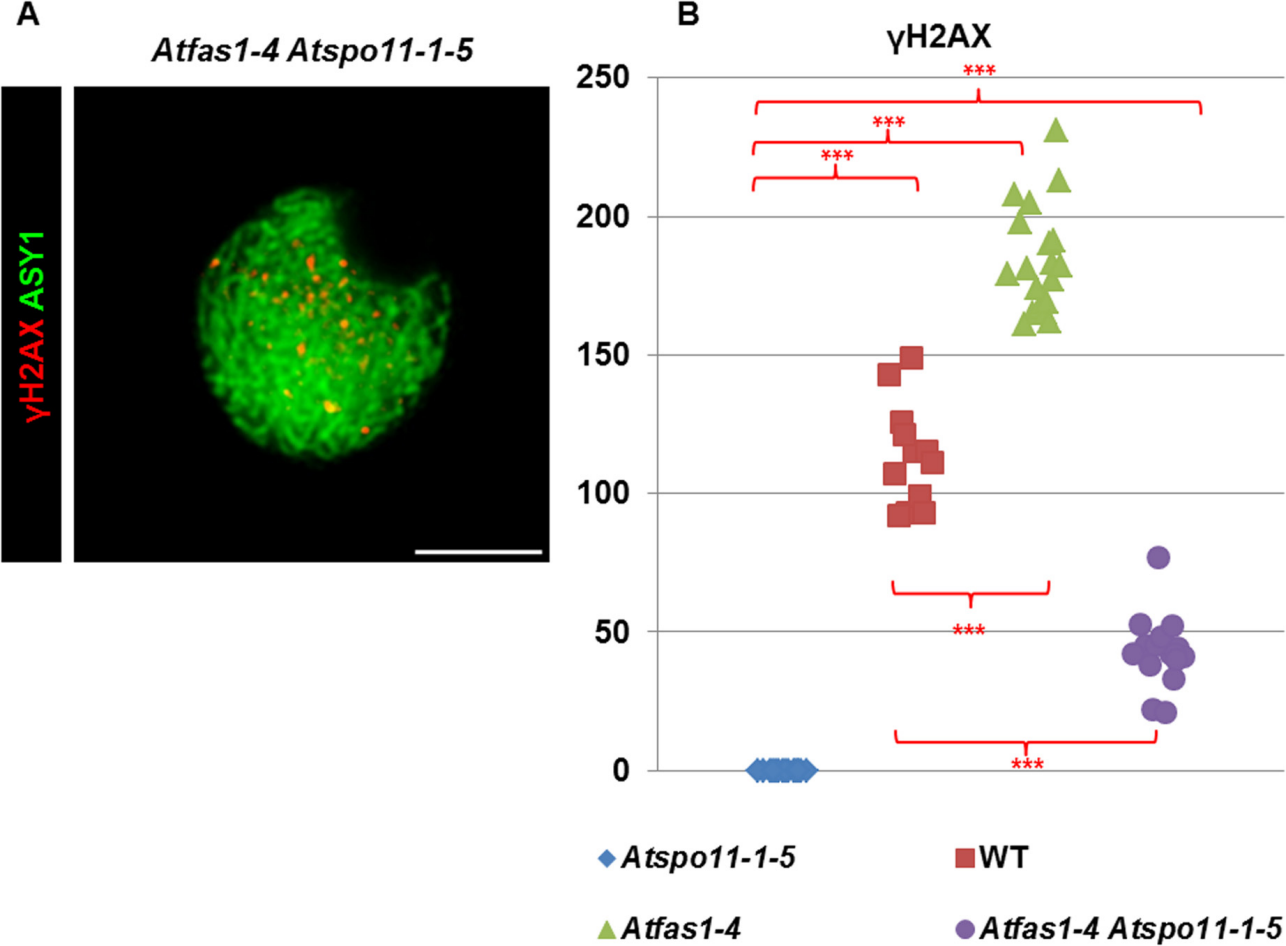
DSB formation in *Atfas1-4*, *Atspo11-1-5*, and in the double mutant *Atfas1-4 Atspo11-1-5*. (A) Dual immunolocalization of AtASY1 (green) and γH2AX (red) in *Atfas1-4 Atspo11-1-5*. Bar = 5 µm. (B) Quantification of γH2AX foci in the different backgrounds. Each dot is the count from a single nucleus. P values are from two-sided Wilcoxon Mann-Whitney tests (***P < 0.001).

DSBs fail to form in *Atspo11-1-5* and as a result, synapsis does not occur (Fig 4A) and ten univalents are observed at diplotene-metaphase I (Fig 4B) leading to unbalanced chromosome segregation after meiosis II (Fig 4C and 4D). However, in the double mutant *Atfas1-4 Atspo11-1-5* we observed very small pairing stretches (Fig 4F, arrow), later confirmed by immunodetection of AtZYP1 (see below), and some chromosome associations at metaphase I (Fig 4G, arrow). Unbalanced segregation at meiosis II and tetrads with unequal sized nuclei were also observed (Fig 4H and 4I). Mean chiasma frequency per cell in the double mutant was 0.11 ± 0.05 (n = 45) and in 11% of the cells at least one chiasma was detected (Fig 4J). These results suggest that some DSBs are being produced in *Atfas1-4 Atspo11-1-5*. We further investigated the level of these residual DSBs by immunolocalization of γH2AX (Fig 5A). We observed 42.81 ± 3.18 γH2AX foci (n = 16) in the double mutant, whereas no foci could be detected in the single *Atspo11-1-5* mutant (n = 20; S5 Fig). Fig 5B also illustrates the overall comparison between all the backgrounds analyzed. The number of DSBs was 64% less in the double *Atfas1-4 Atspo11-1-5* mutant than in WT. Thus, it is not possible to restore the meiotic phenotype of *Atspo11-1-5* by deleting AtFAS1. It is also noteworthy that the number of AtSPO11-independent DSBs detected in *Atfas1-4 Atspo11-1-5* (43) is lower than the increase in DSBs observed in the single *Atfas1-4* mutant (180.05 ± 5.7) with respect to WT (114.17 ± 5.29) (S1 Table). This suggests that the additional DSBs produced in *Atfas1-4* may be both AtSPO11-dependent and independent.

#### *Atfas1-4 Atdmc1-2*

*Atdmc1-2* plants fail to synapse at pachytene and have ten univalents at diplotene-zygotene (Fig 4K and 4L). This leads to abnormal segregation at the second division and polyad formation (Fig 4M and 4N). We did not observe any pairing stretches in *Atfas1-4 Atdmc1-2* after DAPI staining (Fig 4P). However, we detected some SC stretches after AtZYP1 immunodetection (see below). We found chiasmatic associations at metaphase I in 14% of the cells analyzed (Fig 4Q). The mean chiasma frequency per cell was 0.13 ± 0.07 (n = 36). The majority of chiasmata occurred between 45S rDNA loci (Fig 4T). Second division was similar to that of *Atdmc1-2* (Fig 4R and 4S). These results show that all *Atfas1-4 Atdmc1-2* DSBs are repaired by an AtDMC1 independent mechanism that is capable of forming some COs.

#### *Atfas1-4 Atrad51-2* and *Atfas1-4 Atrad51-3*

**Fig 6:**
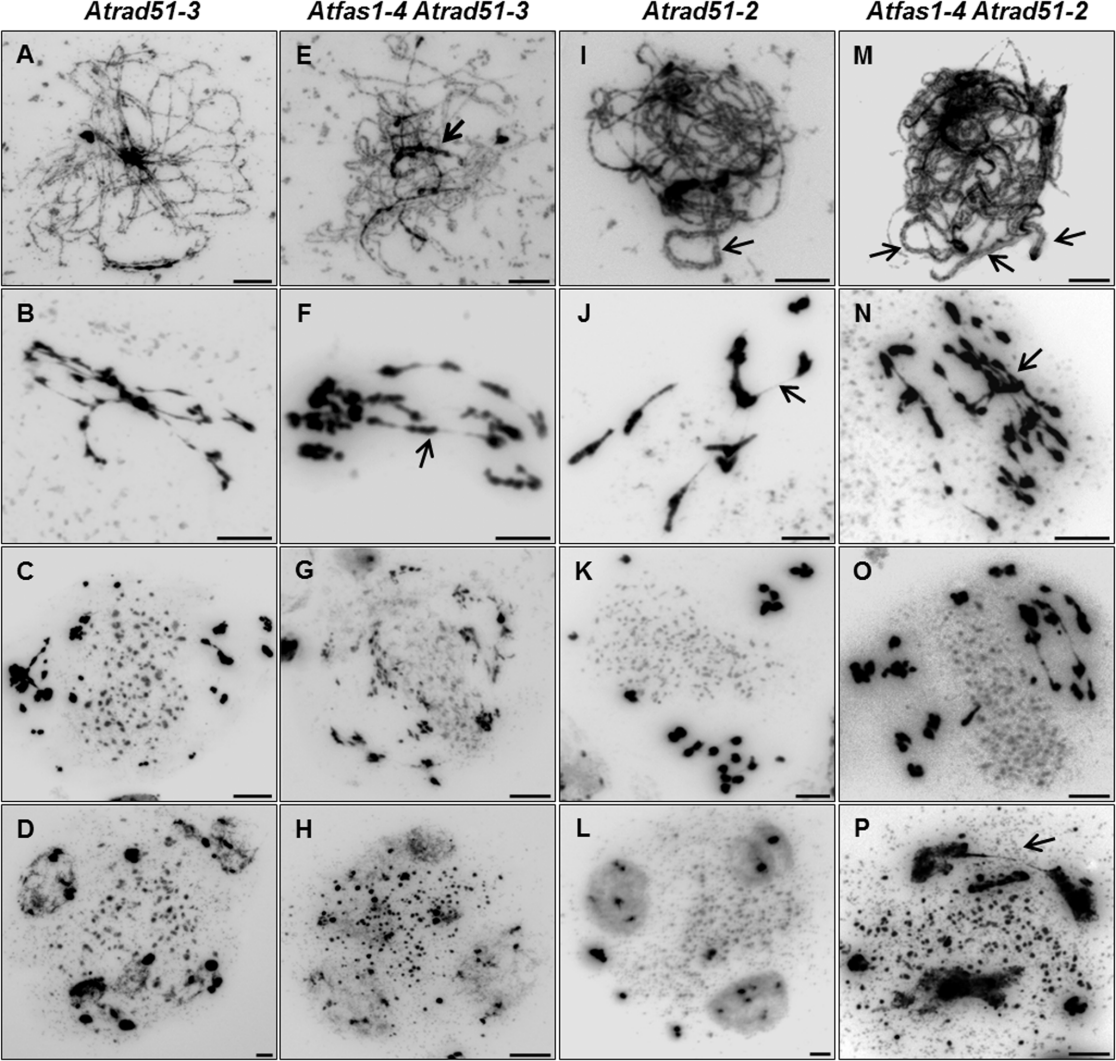
*Atfas1-4 Atrad51* genetic analysis. (A-D) *Atrad51-3* KO mutant plants. Homologous chromosomes do not pair, and severe chromatin fragmentation is observed. (E-H) *Atfas1-4 Atrad51-3*. Certain regions appear to be paired (arrow) at post-leptotene and some associated chromosomes are bioriented at metaphase I (arrow). (I-L) *Atrad51-2* KD mutant plants display some short SC stretches (arrow) and chromosome associations at metaphase I (arrow). (M-P) The double mutant *Atfas1-4 Atrad51-2* also shows short SC stretches (arrows) at post-leptotene and bioriented associated chromosomes at metaphase I (arrow). Bars = 5 µm.

Pairing defects and asynapsis were observed at post-leptotene in *Atrad51-3* (Fig 6A), and chromosomes were entangled and interconnected at metaphase I (Fig 6B). After the second division we observed chromosome/chromatid fragments (Fig 6C) and polyads containing micronuclei (Fig 6D). The meiotic phenotype of *Atfas1-4 Atrad51-3* was slightly different: pairing stretches at prophase I (Fig 6E, arrow) and some conspicuous chromosome associations at metaphase I (Fig 6F, arrow). The number of chromosome/chromatid fragments and polyads observed at second division appeared to be higher than in *Atrad51-3* (Fig 6G and 6H).

*Atrad51-2* meiosis was previously described by Pradillo and colleagues [25]: i) few pairing stretches at post-leptotene (Fig 6I); ii) univalents, well-defined homologous and non-homologous bivalents, multivalents, and small chromosome/chromatid fragments at metaphase I (Fig 6J); iii) chromosomes, chromosome fragments and chromatids lagged in the organelle zone at metaphase II (Fig 6K); and iv) polyads with micronuclei (Fig 6L). *Atfas1-4 Atrad51-2* meiocytes presented partial post-leptotene pairing (Fig 6M). At metaphase I the number of fragments and associations with bioriented chromosomes were higher than in *Atrad51-2* (Fig 6N), and the elevated level of fragmentation persisted into the second division (Fig 6O). The number of nuclei in polyads was also higher than in the single mutant (Fig 6P).

**Fig 7:**
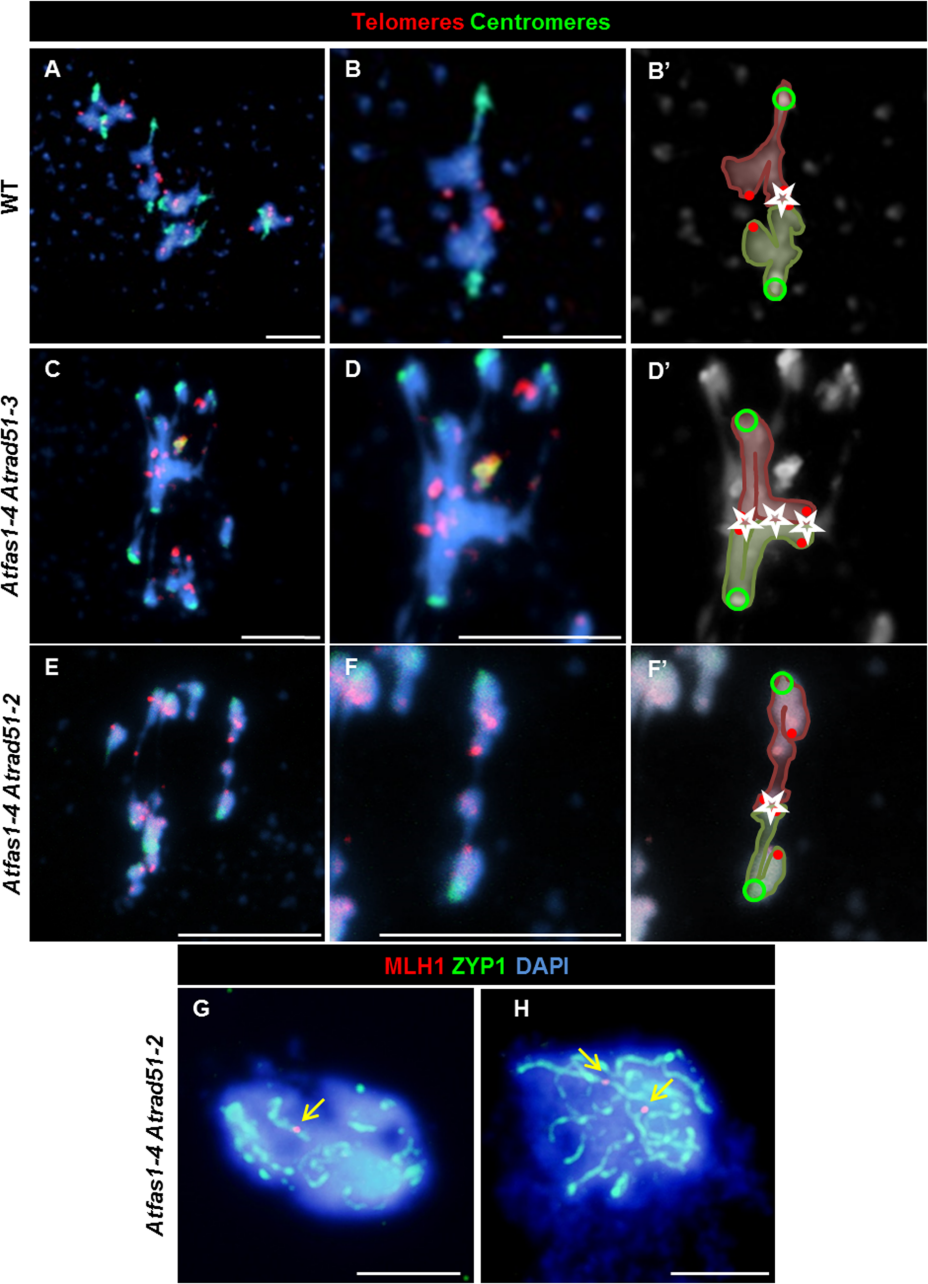
*Atrad51* mutants display truly COs. (A-F) FISH of metaphase I spreads of WT, *Atfas1-4 Atrad51-3* and *Atfas1-4 Atrad51-2* PMCs using telomeric (red) and centromeric (green) probes. (A) WT metaphase I. (B, B’) Magnification of one bivalent from (A). The external localization of the telomeres (red dots) shows a chiasmatic association (star) between two bioriented homologous chromosomes (centromeres are represented as green circles). Representative images of metaphase I cells from (C) *Atfas1-4 Atrad51-3* and (E) *Atfas1-4 Atrad51-2*. (D, D’, F, F’) Bivalent magnification and diagrammatic chiasma interpretation. (G, H) CO confirmation (arrow) in *Atfas1-4 Atrad51-2* detected by immunolocalization of AtMLH1 (red) and AtZYP1 (green) and counterstained with DAPI (blue). Bars = 5 µm.

The centromeric tension and orientation of chromosomes towards opposite poles observed in *Atfas1-4 Atrad51-2* suggests the existence of chiasmatic associations at metaphase I (Fig 6N). To address this question, we carried out FISH analysis with telomere and centromere probes to confirm the nature of these associations. The presence of an unlabeled DAPI stained strip running longitudinally through the center of the terminal associations and the telomeric signals located in the outer part of chromosome junctions constitute cytological evidence of subterminal chiasmata (Fig 7A–7F) (see for discussion [25, 26]). Visualization of AtMLH1 foci provided additional evidence that these associations are chiasmata (Fig 7G and 7H). On these grounds, the mean chiasma frequency per cell in *Atfas1-4 Atrad51-2* plants was 2.08 ± 0.19 (n = 30), which is significantly higher than the result obtained for *Atrad51-2* 1.55 ± 0.09 (n = 30; t = 2.54; P = 0.01). Likewise, both values are significantly higher than the mean observed in *Atfas1-4 Atrad51-3*, 0.91 ± 0.15 (n = 27) (t = -5.18; P < 10^−4^, and t = -3.78; P = 4.16 x 10^−3^, respectively).

### SC formation

To determine if the small pairing patches detected by DAPI staining were truly SC stretches we used immunodetection of AtZYP1. We estimated two different parameters (Tables 3 and 4 and S3 and S4): i) we quantified the number of synaptic initiation points (SIPs) per cell in PMCs that had AtZYP1 signal covering at most 10% of the total of the chromosome axis; ii) we measured the total SC length in PMCs that had AtZYP1 signal covering all the chromosome axes. The 10% criterion was not applied to *Atspo11-1-5*, *Atdmc1-2*, *Atrad51-3*, *Atfas1-4 Atspo11-1-5* and *Atfas1-4 Atdmc1-2* because synapsis rarely goes beyond this percentage.

**Table 3:** Mean numbers of SIPs per cell in the mutants analyzed.

	Mean number of SIPs	Range	n
**Col**	8.89 ± 1.46	3–18	12
***Atfas1-4***	9.54 ± 1.12	4–16	11
***Atspo11-1-5***	1.65 ± 0.29	0–4	27
***Atfas1-4 Atspo11-1-5***	13.54 ± 1.82	3–24	15
***Atdmc1-2***	5.23 ± 0.44	0–14	56
***Atfas1-4 Atdmc1-2***	12.50 ± 1.54	4–23	15
***Atrad51-3***	8.07 ± 0.55	0–18	57
***Atfas1-4 Atrad51-3***	13.89 ± 1.15	1–26	26
***Atrad51-2***	9.57 ± 1.12	0–28	30
***Atfas1-4 Atrad51-2***	18.07 ± 1.16	4–28	30

**Table 4:** Comparison of SC lengths among different mutants.

	SC mean length (µm)	Range	Maximun percentage of synapsis observed	n
**Col**	165.40 ± 4.79	140.35–188.77	100	20
***Atfas1-4***	160.67 ± 8.32	143.60–183.50	100	12
***Atspo11-1-5***	1.15 ± 0.28	0–4.63	2.89	27
***Atfas1-4 Atspo11-1-5***	6.98 ± 0.55	1.83–10.82	6.54	24
***Atdmc1-2***	2.66 ± 0.28	0–10.77	6.50	56
***Atfas1-4 Atdmc1-2***	5.89 ± 2.29	0.37–29.80	18.03	15
***Atrad51-3***	5.35 ± 0.60	0–25.62	15.50	57
***Atfas1-4 Atrad51-3***	14.64 ± 1.77	2.59–35.34	21.39	28
***Atrad51-2***	9.57 ± 1.74	0–38.35	23.21	30
***Atfas1-4 Atrad51-2***	40.67 ± 4.97	2.36–114.16	68.98	30

**Fig 8:**
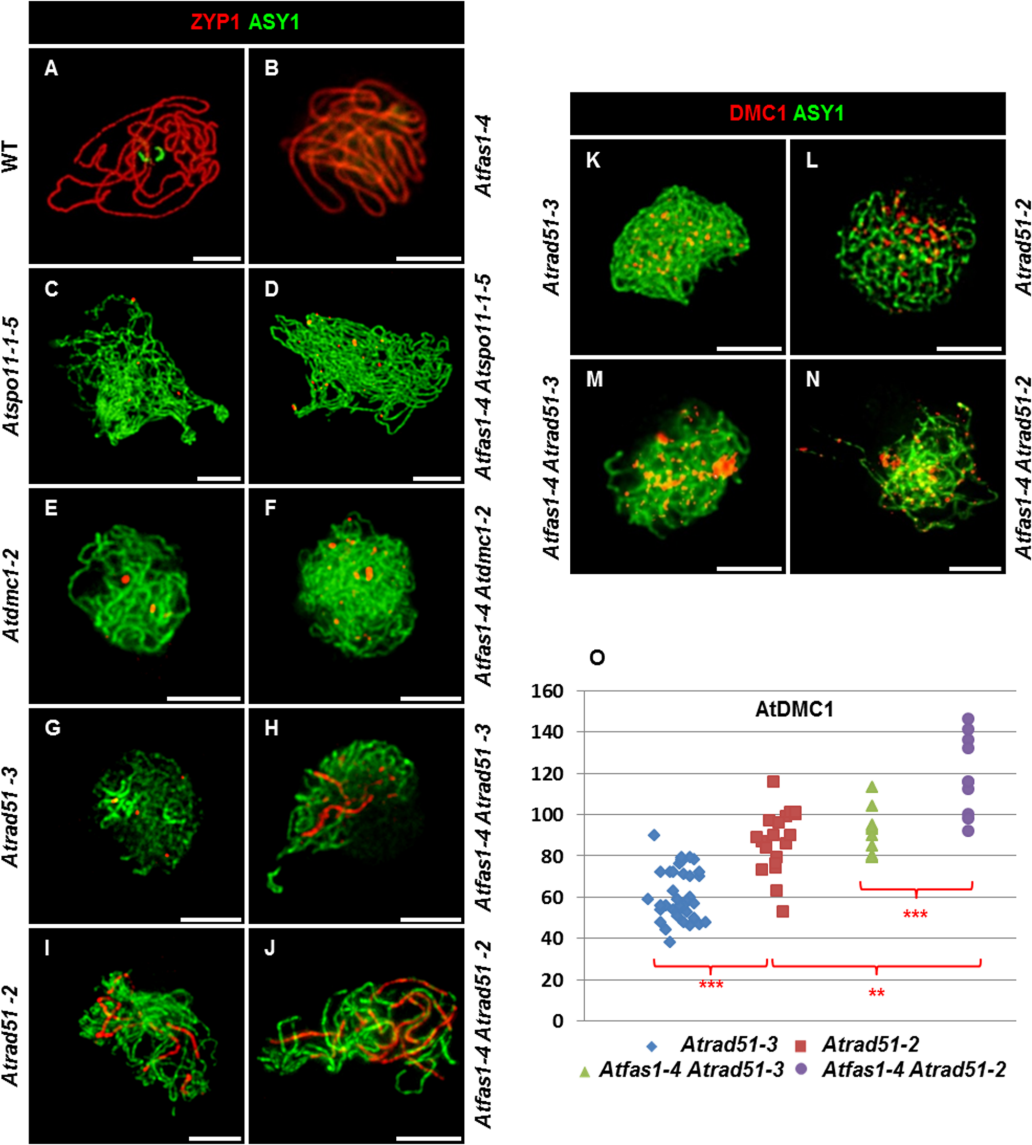
AtRAD51 and AtDMC1 could play different roles in the synaptic process. (A-J) Dual immunolocalization of AtASY1 (green) and AtZYP1 (red) on (A) WT, (B) *Atfas1-4*, (C) *Atspo11-1-5*, (D) *Atfas1-4 Atspo11-1-5*, (E) *Atdmc1-2*, (F) *Atfas1-4 Atdmc1-2*, (G) *Atrad51-3*, (H) *Atfas1-4 Atrad51-3*, (I) *Atrad51-2* and on (J) *Atfas1-4 Atrad51-2*. Dual immunolocalization of AtASY1 (green) and AtDMC1 (red) on (K) *Atrad51-3*, (L) *Atrad51-2*, (M) *Atfas1-4 Atrad51-3*, and on (N) *Atfas1-4 Atrad51-2*. Bars = 5 µm. (O) Dispersion diagram for the total foci per nucleus in *Atrad51-3* (blue), *Atrad51-2* (red), *Atfas1-4 Atrad51-3* (green), and *Atfas1-4 Atrad51-2* (purple) PMCs are indicated. Each dot is the count from a single nucleus. P values are from two-sided Wilcoxon Mann-Whitney tests (**P < 0.01; ***P < 0.001).

Both WT (Fig 8A) and *Atfas1-4* (Fig 8B) achieved full synapsis at pachytene. The results obtained for the different double mutants can be summarized as follows:

#### *Atfas1-4 Atspo11-1-5*

*Atspo11-1-5* had the lowest number of SIPs and the shortest SC length (Fig 8C). However, in *Atfas1-4 Atspo11-1-5* the number of SIPs was restored to that observed in WT (8.89 ± 1.46; Fig 8D). This is reflected in the significant increase in synapsis extension observed in the double mutant (2.89% in *Atspo11-1-5 vs*. 6.54% in *Atfas1-4 Atspo11-1-5*). Hence, AtSPO11 independent DSBs are apparently being processed by the meiotic machinery, as shown by the residual COs detected.

#### *Atfas1-4 Atdmc1-2*

In *Atdmc1-2* the number of SIPs was very low and SC formation very limited (Fig 8E). In *Atfas1-4 Atdmc1-2* the number of SIPs was restored to normal levels, but not SC extension (Fig 8F). It is noteworthy that both the mean and the maximum SIP values in these mutants were lower than those defective for AtRAD51.

#### *Atfas1-4 Atrad51-3*

No differences in the number of SIPs were found between *Atrad51-3* (Fig 8G) and WT, although, at most 15.5% of the complement was synapsed in this mutant. The mean length of AtZYP1 signals was similar between *Atrad51-3* and *Atfas1-4 Atdmc1-2*. In contrast, both mutants have significantly shorter SCs than *Atfas1-4 Atrad51-3* (Fig 8H).

#### *Atfas1-4 Atrad51-2*

The mean number of SIPs in *Atrad51-2* (Fig 8I) was similar to WT, but some PMCs showed more than 20 SIPs. The maximum SC length observed increased up to 23.2%. Consequently, the proportion of the chromosome complement that achieved synapsis in *Atfas1-4 Atrad51-2* (Fig 8J) was longer than in *Atfas1-4 Atrad51-3* (Fig 8H) and increased up to 68.98%.

### Immunolocalization of AtDMC1

The dynamics of synapsis described above suggests that the differences between mutants might be due to variation in the number of DSBs formed and/or the balance between RAD51 and DMC1 during early prophase I. To test this, we measured the number of AtDMC1 foci in mutants defective for AtFAS1 and AtRAD51.

There was a significant increase in the number of leptotene AtDMC1 foci in *Atfas1-4 Atrad51-3* (Fig 8M) and *Atfas1-4 Atrad51-2* (Fig 8N) when compared to the respective single mutants (Fig 8K and 8L and S1 Table). Also, the mean number of AtDMC1 foci in PMCs of *Atrad51-3* was significant lower than in *Atrad51-2* (W = 183.5; P = 3.30 x 10^−6^), and between *Atfas1-4 Atrad51-3* and *Atfas1-4 Atrad51-2* (W = -40.0; P = 2.81 x 10^−3^), as well as between *Atrad51-3* and *Atfas1-4 Atrad51-*2 (W = 190.0; P = 1.47 x 10^−7^) (Fig 8O). However, there were no differences between *Atrad51-2* and *Atfas1-4 Atrad51-3* (W = 13.5; P = 0.567). Among all of these genotypes, *Atfas1-4 Atrad51-2* showed the highest number of AtDMC1 foci but it was always lower than those in WT and *Atfas1-4* (W = 51.5; P = 0.046 and W = 89.0; P = 2.17 x 10^−5^, respectively).

## Discussion

### Meiotic DSB frequency is increased in *Atfas1-4*

Reciprocal recombination events (COs) between homologous chromosomes are necessary in many organisms to ensure correct chromosome segregation at first meiotic division. This is accomplished using highly regulated pathways initiated by programmed DSBs catalyzed by SPO11 (S4 Fig, [27]). Typically, an excess of DSBs are generated, and only a small fraction mature as COs (cytologically manifested as chiasmata), the rest being repaired by other pathways. There are differences in the DSBs/CO ratio among species [28]. Neither physical genome size nor chromosome number can explain these differences. Budding yeast has about two times more DSBs than COs [29], while Arabidopsis has approximately 15 times more DSBs than COs [30, 31]. In Arabidopsis the number of DSBs can be estimated using recombination protein foci (e.g. γH2AX, AtRAD51 and AtDMC1) as a proxy. As a result, different studies, using different antibodies for immunolocalization have estimated different numbers [see 30, 32–36]. Nevertheless, using the same technique it is possible to compare WT with different mutant backgrounds accurately.

The number of γH2AX, AtRAD51 and AtDMC1 foci in *Atfas1-4* was significantly higher than in WT (Fig 2 and S1 Table). The most increase of DSBs might be a consequence of breaks produced during pre-meiotic DNA replication as occurs in the somatic line [17]. In fact, AtRAD51 foci appeared in *Atfas1-4* earlier than in WT (Fig 2E and 2F), in a stage (pre-meiotic G_2_) when DSBs catalyzed by AtSPO11 have not yet been produced. These DSBs are likely repaired by HR with the sister chromatid serving as template, since AtDMC1 is unavailable at G_2_. Alternatively, the increase in DSBs may be related to a more open chromatin configuration exhibited by the mutant that may facilitate access by topoisomerases (S3 Fig; [18]). The residual DSBs detected by immunolocalization of γH2AX (Fig 5A) in *Atfas1-4 Atspo11-1-5* and its comparison with those observed in the single mutant *Atfas1-4* (Fig 2C) demonstrate that the increase in DSBs produced in *Atfas1-4* most likely involves both mechanisms. Furthermore, the analysis of the double *Atfas1-4 Atspo11-1-5* mutant revealed that a portion of these AtSPO11 independent DSBs may be processed as genuine programmed DSBs, similar to those produced by cisplatin [30], since, in contrast to *Atspo11-1-5*, chiasmata and SC were observed. Another conclusion that comes from this result is that a minimum number of DSBs is required to guarantee full synapsis and the obligatory CO. According to γH2AX immunolocalization, the number of DSBs in *Atfas1-4 Atspo11-1-5* (42.81 ± 3.1) represents less than the 40% of the total DSBs produced in WT (114.17 ± 5.29). However, this number is enough to restore a normal SIPs phenotype (Fig 8D and Table 3). These findings could indicate that SC nucleation and elongation are dependent on the number of DSBs, which in *Atfas1-4 Atspo11-1-5* are insufficient to achieve both full synapsis and the obligate CO [37, 38]. In this context, we cannot exclude the possibility that the excess of DSBs in *Atfas1-4* derives from a delayed prophase I.

### “Extra” DSBs of *Atfas1-4* are repaired by HR

At least four genes involved in HR are overexpressed in this mutant: *AtBRCA1*, *AtCOM1*, *AtRAD51* and *AtDMC1* (Fig 2A). AtBRCA1 loads recombinases on to DNA [39, 40]. The expression of this gene depends on DNA damage, for instance Lafarge and Montané [41] reported that a 100 Gy dose produces about 92-fold overexpression of this gene. In mammals BRCA1 interacts with MRN (MRE11-RAD50-NBS1), CtIP (COM1-SAE2), and Retinoblastoma, to activate G_2_ cell cycle check-point by means of ATM phosphorylation [42, 43].

In *Atcom1-1* there is an accumulation of AtSPO11 at prophase of meiosis I without formation of AtRAD51 foci, despite the presence of unrepaired DSBs. AtCOM1 acts downstream of AtSPO11-1 and upstream of AtBRCA1 and AtDMC1. Thus, it is necessary for regular turnover of AtSPO11-1 and processing of meiotic DSBs [44]. Absence of AtCOM1 causes chromosome fragmentation like other mutants defective in creation of 3’ ssDNA tails via resection [45, 46]. Furthermore, DNA fragmentation in the mutant is suppressed by eliminating AtSPO11-1 [44]. The overexpression of AtCOM1, observed in *Atfas1-4*, might be facilitating the correct processing (resection) of the extra DSBs.

Once 3’ ssDNA tails are generated at the DSB site, RAD51 and DMC1 catalyze a strand exchange reaction with an intact DNA duplex to produce joint molecule intermediates (see S4 Fig; [47]). These recombinases bind the ssDNA tails to produce presynaptic filaments. It has been proposed that DMC1 is essential to establish preferred interactions between homologs, whereas the catalytic activity of RAD51 alone results in sister chromatid exchanges [28, 30, 35, 48–50]. However, the exact action of both recombinases is not fully understood (see [27] for review in Arabidopsis). *AtDMC1*, as well as *AtCOM1* and *AtBRCA1*, are about 3-fold overexpressed in *Atfas1-4*. However, *AtRAD51* is about 5-fold overexpressed (Fig 2A), an increase that could be related to the extra AtSPO11-independent DSB repair, in concordance with the early meiotic appearance of AtRAD51 in *Atfas1-4* compared to WT (Fig 2E and 2F). Increases of *AtRAD51* and *AtDMC1* mRNA were accompanied with those of their respective protein products (Fig 2I and 2L). The abundance of these proteins may help prevent chromosome fragmentation despite an increase in DSBs. The overexpression of *AtSMC6* in the mutant could be also related to the repair of non-programmed DSBs [51–53]. Accordingly, it is tempting to speculate about the existence of an increase in inter-sister recombination. On the other hand, although the most likely explanation for the overexpression of HR genes is the requirement for repairing the “extra” DSBs, another possibility is an extended prophase I in *Atfas1-4*. Since only some genes are overexpressed, a global effect on transcription because of changes in chromatin structure does not seem to be probable.

### Increase of GC events in *Atfas1-4*

COs can be produced by at least two pathways in Arabidopsis. Type I COs are subject to positive interference and are dependent on the ZMM proteins [54–56]. Type II COs are insensitive to interference, are randomly distributed and are dependent on MUS81 and MMS4 proteins for their formation [57, 58]. In *Atfas1-4*, genes involved in either Type I COs (*AtMLH3*) or Type II COs (*AtMUS81*) were not overexpressed (Fig 2A). Likewise, the number of both AtMLH1 (Type I) and AtMUS81 foci (Type II) was similar to WT (Fig 2R and 2U). These results are consistent with the absence of differences in chiasma frequency between the mutant and WT (Table 1). According to the model proposed by Berchowitz and Copenhaver [59], if an increase in DSBs is not translated to a CO increase it implies that there is an increase either in the number of recombination intermediates in the Synthesis Dependent Strand Annealing (SDSA) pathway or in the frequency of double Holliday Junctions (dHJs) resolved as NCOs (S4 Fig). Although during meiosis there is a bias against inter-sister events, an alternative explanation is an increase in inter-sister repair events [60].

AtMSH4 and AtMSH5 form a complex that loads and stabilizes dHJs [61–63]. Since the number of AtMSH4 foci was similar in *Atfas1-4* and WT (Fig 2O), we conclude that an overall change in the number AtMSH4 foci did not take place. Cole et al. [64] showed that mice that over-express SPO11β (2X higher expression) have twice the level of γH2AX, whereas they have only 7% more MSH4. In *Atfas1-4* an increase of 58% in DSBs does not change either the number of AtMSH4 foci (Table 1) or the number of SIPs (Tables 3 and S3). However, since our observations correspond to mid-late zygotene we cannot exclude a possible increase of AtMSH4 foci in early stages. Additionally, it is interesting to note that mRNA levels of *AtBLAP75* and *AtTOP3α* have suggestive (but not statistically significant) increases in expression levels, and that both are involved in dHJ dissolution leading to NCOs [65, 66].

Sun and colleagues [24] reported differences in the GC frequency at several loci in WT, for instance GC events were most frequent in the short arm of chromosome 4 followed by those produced in chromosomes 2 and 1 (4 > 2 >1). The situation in *Atfas1-4* is exactly the opposite since the magnitude of GC events was 1 > 2 > 4 (Table 2). It seems that in *Atfas1-4* those regions that are less prone to GC in WT are more prone to processing excess DSBs with a mechanism that also yields GCs. Local specific changes in chromatin conformation could also affect these GC frequencies.

Martini and colleagues [67] reported in yeast that a decrease in the number of DSBs observed in different *spo11* alleles did not affect the overall CO frequency at the expense of a decrease in the number of NCO events. This phenomenon was called “CO homeostasis”. On the other hand, in mice overexpressing *SPO11* the mean number of RAD51 and DMC1 was higher than in WT, whereas MLH1 foci number was similar [65]. Our results are in some way similar to these because the increase in the number of DSBs observed in *Atfas1-4* had no influence on the frequency of COs (scored as either chiasmata or AtMLH1/AtMUS81 foci), or on their distribution (Figs 2P–2U and 3). However, the excess of DSBs produces an increase in the frequency of GC events. We cannot exclude that the increase in GC frequency could be due to mismatch repair defects or alterations in inter-homolog/inter-sister interactions. Nevertheless, the existence of CO homeostasis, as a consequence of positive CO interference, is a likely explanation. Joshi et al. [68] have recently demonstrated that recombination outcome is dependent on global DSB levels. Thus, early and low-abundant DSBs lack homolog bias. This could be the case for pre-meiotic G_2_ DSBs observed in *Atfas1-4*. Following the model proposed by Joshi et al. [68], the gradual implementation of interhomolog bias would produce an increase in interhomolog interactions throughout prophase I in *Atfas1-4*, with an increase in NCOs *vs*. CO compensating for increased homolog bias.

### The interplay between AtRAD51 and AtDMC1 in *Atfas1-4*

The double mutants *Atfas1-4 Atrad51-2*, *Atfas1-4 Atrad51-3*, and *Atfas1-4 Atdmc1-2* enabled functional analysis of the interaction between AtRAD51 and AtDMC1. An increase in DSBs in absence of AtFAS1 is accompanied by an excess of AtDMC1 in *Atfas1-4 Atrad51*, and AtRAD51 in *Atfas1-4 Atdmc1-2*. Bishop and colleagues [69] and Tsubouchi and Roeder [70] demonstrated in yeast that high levels of inter-homolog recombination could be achieved in the absence of Dmc1 upon either overexpression of *Rad51* or by stimulating its partner *Rad54*. Recently, Cloud and colleagues [48] reported that the activity of Dmc1, but not that of Rad51, is necessary for meiotic recombination in yeast. Thus, Rad51 may be an accessory factor which contributes to homolog bias independently of strand exchange activity. However, it may also be relevant when Dmc1 fails. On the other hand, Hong and colleagues [71] and Lao and colleagues [72] have provided evidence for the inhibitory role of Dmc1 in Rad51 activity, and that the default option for recombination is homolog bias, independent of whether strand exchange is promoted by either DMC1 or RAD51. Observations of da Ines and colleagues [50] in Arabidopsis support an accessory role of AtRAD51. However, Pradillo and colleagues [25] observed homologous and non-homologous recombination in a KD *Atrad51* mutant, which also had less chromosome fragmentation than the KO mutant. These results suggest that some DSBs can be processed by interhomolog recombination in the mutant, although a critical level of this protein is required to ensure the fidelity of HR during inter-chromosomal exchanges. On the other hand, Kurzbauer and colleagues [35] have reported that *Atrad51* is defective in AtDMC1 loading. Likewise, Uanschou and colleagues [73] have pointed out that AtDMC1 might have a role as an inhibitor of AtRAD51 [27].

*Atfas1-4 Atdmc1-2* displayed a similar meiotic phenotype compared to the single mutant *Atdcm1-2* (Fig 4, [74]). However, a few bivalents were observed at metaphase I (Fig 4T). Thus, AtRAD51 is capable of repairing the excess DSBs produced in *Atfas1-4*, and may also permit the formation of some COs independently of AtDMC1. Nevertheless, the presence of other regulatory factors involved in the decision dictating sister chromatid *vs*. inter-homolog exchange cannot be ruled out.

On the other hand, decreased (*Atrad51-2*) or elimination (*Atrad51-3*) of AtRAD51 leads to chromosome fragmentation (Fig 6), the effects being more drastic in *Atfas1-4 Atrad51-3* than in *Atfas1-4 Atrad51-2*. These results constitute additional evidence of excess DSBs in *Atfas1-4*. In these double mutants, the number of AtDMC1 foci was higher than in *Atrad51* but lower than WT because, despite AtDMC1 overexpression, AtRAD51 is necessary to correct AtDMC1 loading on chromosome axes [32, 35]. The presence of a certain amount of AtRAD51 in *Atfas1-4 Atrad51-2* promoted COs dependent on AtMLH1. Moreover, the fact that chiasma frequency was higher in *Atfas1-4 Atrad51* than in *Atfas1-4 Atdmc1-2* revealed the important role of AtDMC1 in homologous interchanges. This suggests that AtDMC1 is more efficient than AtRAD51 in promoting homologous interchanges [75].

### AtRAD51, AtDMC1 and SC formation

The relationship between synapsis initiation at recombination initiation sites and its ultimate progression varies depending on species, sex, and other factors [37, 61]. In Arabidopsis, there are differences in chiasma frequency and SC length between PMCs and megaspore mother cells (MMCs) of the same plant, and between different accessions [23, 76–78]. SC length is also determined by genome size, and in Arabidopsis this relation, measured as cM/Mb, is the highest described in any plant species to date [23]. However, the chromatin decondensation exhibited by *Atfas1-4* did not imply any changes in either the initial SC nucleation or total SC length (Tables 3 and 4).

In yeast and mouse, MSH4 localizes over SIPs and it has been proposed that this protein cooperates with RAD51 and DMC1 in SC nucleation [79, 80]. In *Atfas1-4* as well as in WT plants, the number of AtMSH4 and AtMSH5 foci estimated by immunolocalization greatly exceed that of SIPs and COs (Table 3; [61–63]). This fact maybe explained by the necessity of establishing efficient homologous interactions at prophase I, not necessarily essential to achieve full synapsis [61–63, 81]. The increase of DSBs in *Atfas1-4*, either dependent or independent of AtSPO11, did not produce changes in the frequency of SIPs, SC length or the number of late AtMSH4 foci. As we discussed above, less than the 40% of the total DSBs produced in WT are enough to restore a normal number of SIPs in *Atfas1-4 Atspo11-1-5* (Fig 8D and Table 3). However, while there were not differences in the number of SIPs between *Atrad51-3* and WT, *Atdmc1-2* displayed lower values (Fig 8 and Tables 3 and S3). Thus, both recombinases are probably able to catalyze HR and to be involved in the SC initiation as occurs in yeast [73, 82, 83], although AtDMC1 seems to be the most efficient. The normal number of SIPs was restored in *Atfas1-4 Atdmc1-2*, but in *Atfas1-4 Atrad51-3* the mean number of SIPs greatly exceeded normal levels [23, 76–78].

In any of the mutants analyzed, AtZYP1 localized along the entire length of AEs, indicating that both recombinases are indispensable for achieving full synapsis. However, the presence of only one of them generated differences in synapsis progression rates. Thus, there were no differences in SC length between *Atdmc1-2* and *Atfas1-4 Atdmc1-2*, but they existed between *Atrad51-*3 and *Atfas1-4 Atrad51-3*. A possible interpretation of these results is that although *AtRAD51* overexpression does not produce changes in SC lengths, AtDMC1 does (Fig 8 and Tables 4 and S4). AtDMC1 activity may also be favored by a certain amount of AtRAD51 because the number of SIPs and SC length are higher in *Atfas1-4 Atrad51-2* than in *Atfas1-4 Atrad51-3*, perhaps due to AtRAD51 contribution to AtDMC1 loading on chromosome axes (Fig 8; [32, 35]). Finally, it is noteworthy that in all mutants analyzed there was a positive relationship between the number of AtDMC1 foci and the development of the synaptic process. That is, the higher number of AtDMC1 signals the higher the number of SIPs and the longer the SC length (Fig 8, S1 and S3 and S4 Tables). Another issue to consider for the interpretation of this result is the balance between both recombinases, which influences correct SC assembly [30, 35]. In *Atfas1-4 Atrad51-2*, *AtDMC1* is overexpressed and there is presumably more AtRAD51 than in the single mutant *Atrad51-2*. These observations could reflect the interplay between control of chromosome axes and HR, a system in which AtASY1, AtASY3 and AtDMC1 would have similar roles to Hop1, Red1 and Dmc1 in yeast [30, 33, 84].

### Concluding remarks

**Fig 9:**
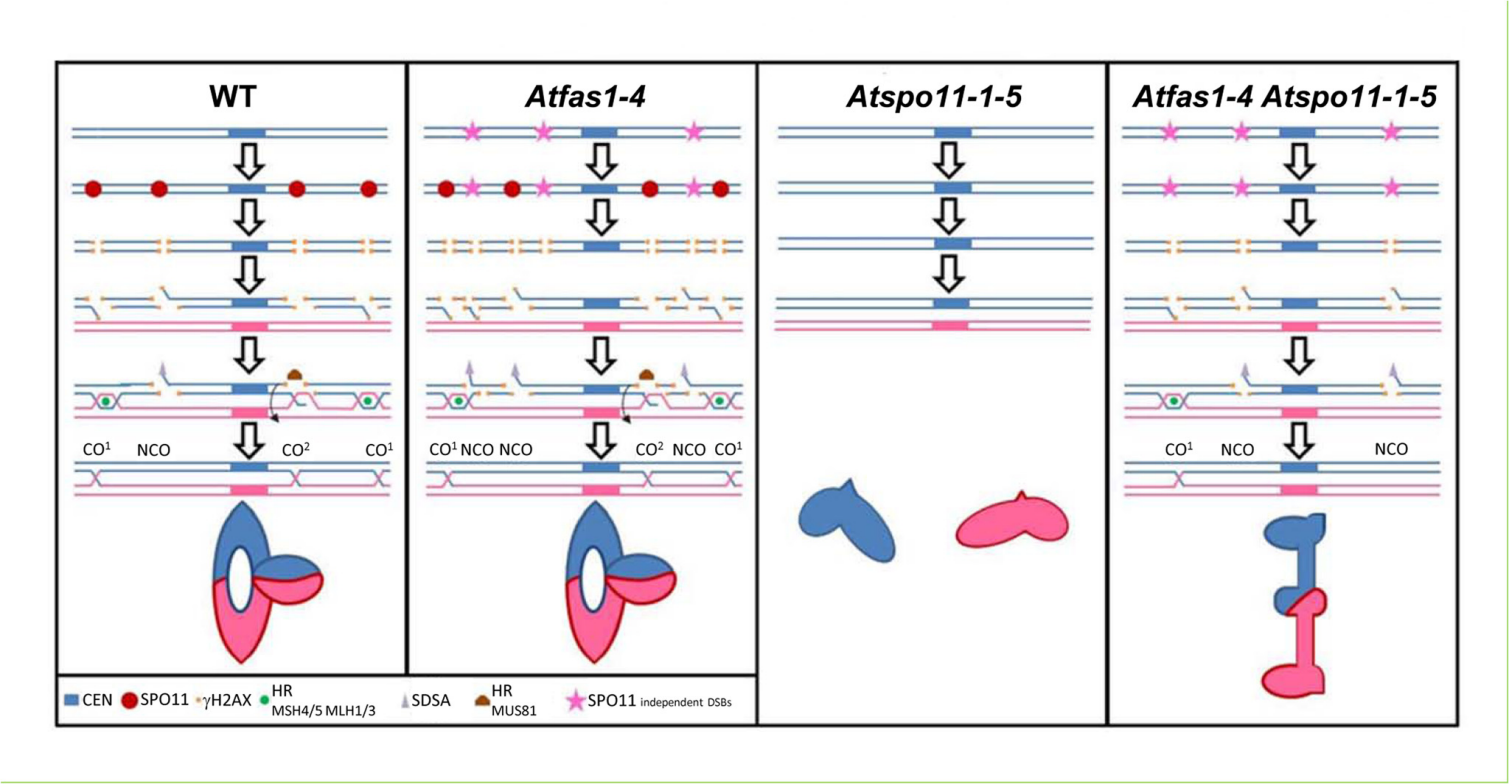
Meiotic DNA DSB outcomes in Arabidopsis. The model shows the different meiotic fates of DSBs (AtSPO11-dependent and independent) in WT, *Atfas1-4*, *Atspo11-1-5*; and in the double mutant *Atfas1-4 Atspo11-1-5*. (CEN: centromere; HR: Homologous Recombination; SDSA: Synthesis Dependent Strand Annealing).

Summing up, our results show that the number of COs can be constrained in plant species even when the number of DSBs increases during meiosis. Fig 9 shows different models for this regulation in the different backgrounds analyzed. In WT meiosis, DSBs catalyzed by AtSPO11 can be processed by different pathways; mainly by HR producing mostly COs and possibly some NCOs, or by SDSA producing NCOs. In absence of AtSPO11-catalyzed DSBs, there is no HR, no COs are formed and the homologous chromosomes are not linked by chiasmata at metaphase I. In a double mutant *Atspo11-1-5 Atfas1-4*, AtSPO11-independent DSBs can be processed as NCOs and COs producing some bivalents at metaphase I. Thus, some AtSPO11-independent DSBs can be processed by HR to form COs and produce chiasmata between homologous chromosomes. In the single *Atfas1*-4 mutant, where AtSPO11-dependent DSBs and extra AtSPO11-independent DSBs are formed, the number of COs at metaphase I is the same that in the WT. The extra DSBs appear to be processed to NCOs in this mutant in order to keep the same CO frequency compared to the WT, producing an increase in GC frequency. Taken together, these results highlight the complex regulation of CO formation in Arabidopsis meiosis.

#### WT CO model

The model shows the meiotic processing of AtSPO11-dependent DSBs using two homologous chromosomes (blue and pink). AtSPO11 (red circle) binds to the chromatin along the chromosomes and produces DSBs. The phosphorylation of H2AX (γH2AX, orange squares) occurs rapidly around the DSB chromatin region. The DSBs are processed by the MRN complex that resects the 5’ ends of the DSBs allowing the 3’ ends to be exposed as single strand DNA (ssDNA) tails which could follow different pathways: HR (green circle, CO^1^), SDSA (grey triangle) or AtMUS81 interference-independent CO pathway (CO^2^). During HR the 3’ SSD tails will associate to AtRAD51 and AtDMC1. These recombinases are involved in the ssDNA invasion to search for the homologous sequences to process the DSBs with the homologous chromosome. If they are successful, they will form different recombination intermediates using proteins like AtMSH4/5 and AtMLH1/3 that will end into a double Holliday Junction (dHJ) which could have different outputs: i) dHJ resolution, producing mostly CO^1^ and very little NCOs. These COs are subjected to interference. ii) dHJ dissolution which using proteins like BLAP75 and TOP3α would produce NCOs. iii) The ssDNA tail invasion might not follow the HR pathway and form a dHJ which would be processed by AtMUS81 to produce COs not subjected to interference (CO^2^). iv) If the ssDNA tail invasion is not stable and the 3’ end gets liberated from the initial intermediate with the homologous chromosome, SDSA could process this DSB and repaired it producing a NCO. A metaphase I ring bivalent configuration bearing one chiasma in one arm and two chiasmata in the other is showed.

#### *Atfas1-4* CO model

The model shows the meiotic processing of both, the AtSPO11-dependent (red circles) and AtSPO11-independent (pink stars) DSBs present in this mutant. This mutant presents an increase in DSBs visualized by an increase of -γH2AX (orange squares). These extra DSBs could be processed by the different pathways showed in WT but nevertheless, the outcome of these extra DSBs is to produce NCOs. Thus, keeping the amount of COs to the same quantity of that present in the WT even when the number of DSBs is increased (CO homeostasis). A metaphase I ring bivalent configuration bearing three chiasmata is showed.

#### *Atspo11-1-5*

DSBs are not formed and no HR can be achieved. Two univalents are showed at metaphase I.

#### *Atspo11-1-5 Atfas1-4*

This model shows the meiotic processing of AtSPO11 independent (pink stars) DSBs. Lacking AtSPO11-1 means that not meiotic DSBs are produced and obviously not recombination between the homologous chromosomes can be achieved. Chromosomes at metaphase I cannot form bivalents and only univalents are observed. Nevertheless, in *Atspo11-1-5 Atfas1-4* some bivalents can be formed. In this double mutant AtSPO11-dependent DSBs are not formed but, due to the lack of AtFAS1 protein, some DSBs are produced (probably during or just after the pre-meiotic S phase due to DNA replication errors). These AtSPO11 independent DSBs can be processed to NCOs and also as COs. A metaphase I rod bivalent bearing one distal chiasma is showed.

## Materials and Methods

### Plant materials

Plants were grown, material harvested and DNA extracted as described previously by Pradillo and colleagues [25]. The *Arabidopsis thaliana* Columbia (Col-0) accession was used as a control. Seeds of most mutants were provided by the Nottingham Arabidopsis Stock Center (Nottingham, UK). Dr. Crisanto Gutiérrez kindly donated *Atfas1-4* (SAIL_662_D10; [4]), *Atfas2-1* [85], and RNAi *Atasf1a Atasf1b* plants with decreased levels of both ASF1A and ASF1B [86]. For double mutant analysis, T-DNA alleles previously described by Pradillo and colleagues [25] were used: *Atspo11-1-5* (SALK_009440), *Atrad51-2*, *Atrad51-3* (SAIL_873_C08) and *Atdmc1-2* (SAIL_170_F08). To score GC in *Atfas1-4* we used three NFTLs: NFTL 567-GC1, NFTL 3411-GC1 and NFTL 424-GC1 [24]. Genotyping of double mutants was performed by PCR using a combination of three primers, one T-DNA specific primer and two specific primers for the corresponding lines (S5 Table).

### Cytological analysis

Observations of gynoecia were carried out using a squash procedure with fixed buds. Sepals, petals, and stamens were dissected from flower buds in 100 µl of acetic carmine solution (Sigma) and the number of ovules was scored. Pollen tetrads were collected by dipping flowers in 10 µl of Pollen Growth Media [24]. Fixation, PMC slide preparation and FISH were carried out as described by Sánchez-Morán and colleagues [21]. The DNA probes used were: 45S rDNA (pTa71, [87]), 5S rDNA (pCT4.2, [88]), centromeres (pAL1, [89]), and telomeres (pLT11, [90]). Microscopy was carried out using an Olympus BX-60 microscope equipped with an Olympus DP71 digital camera controlled by analysis software (Soft Imaging System). Images were analyzed and processed with Adobe Photoshop CS4.

The spreading immunolocalization technique previously described by Armstrong and colleagues [91] was used to detect SC and HR related proteins. Prof. Chris Franklin kindly donated primary antibodies: anti-γH2AX (Ser 139, catalog no. 07–164 Upstate Biotechnology; rabbit, 1:250 dilution), anti-AtASY1 (rat; 1:1000 dilution), anti-AtZYP1 (rabbit; 1:500), anti-AtRAD51 (rabbit; 1:250), anti-AtDMC1 (rabbit; 1:250), anti-AtMSH4 (rabbit; 1:250), and anti-AtMLH1 (rabbit; 1:250) [30, 37, 61, 92–94]. Protein foci were manually counted using the Count tool in Adobe Photoshop CS4. SC length was measured using Image J software (http://rsbweb.nih.gov/ij/).

### RNA extraction and quantitative PCR

Total RNA was isolated from young flower buds using an RNeasy kit (Qiagen). Quantitative PCR was performed with the Fast Start Taq Man Probe Master kit using UPL probes and specific primers designed by the UPL Assay Design Center (http://www.roche-applied-science.com/sis/rtpcr/upl/index.jsp?id=UP030000). See S6 Table for qPCR primers and UPL probe information. Relative quantification of mRNA was calculated using fold variation over a calibrator with the standard curves method [95] after normalization to 18S rRNA as an internal control (Hs99999901_s1; Applied Biosystems, http://www.appliedbiosystems.com). Three experimental replicates of five independent RNA extractions were carried out for each target gene.

### Statistical methods

Statistical comparisons of the number of ovules and data on SC formation and HR were performed using a non-parametric Wilcoxon Mann-Whitney test with a confidence interval of 95%. Statistical analysis of chiasma counts was done as described in Sánchez-Morán and colleagues [22, 23] and López and colleagues [24]. We compared GC frequencies between WT and *Atfas1-4* plants using chi-square tests. To evaluate the significance of relative gene expression differences between mutant and WT plants, 95% confidence intervals were defined for the average expression of each gene.

## Supporting Information

S1 Fig *Atfas1-4* plants display a reduction in the number of ovules per gynoecium. Squash procedure to visualize and estimate the number of ovules per gynoecium (A) on WT and (B) on *Atfas1-4*.
(TIF)

S2 Fig *Atfas1-2* and *AtASF1a/AtASF1b* RNAi mutants show normal meiosis. Chromosome spread preparations from (A, C, E, G) *Atfas1-2* and (B, D, F, H) *AtASF1a/AtASF1b* RNAi PMCs. (A, B) Pachytene. (C, D) Metaphase I: four ring bivalents and one rod bivalent on each. (E, F) Prophase II. (G, H) Tetrad. Bars = 5µm.
(TIF)

S3 Fig *Atfas1-4* displays changes in the chromatin during meiosis. FISH using the centromeric probe pAL1 (180 bp) (A) on WT and (B) on *Atfas1-4* at pachytene. In the mutant the centromeric FISH signal is weaker, less defined and more extended than in WT.
(TIF)

S4 Fig *Arabidopsis thaliana* HR model. Single strands of DNA are shown as either blue (parent 1) or red (parent 2) rods. AtSPO11 helped by other proteins initiates programmed DSBs. H2AX phosphorylation occurs at the break zone and DSBs are resected 5’ to 3’ to produce single ssDNA tails by the MRN complex and COM1. One of these ends invades the homologous duplex DNA, giving raise a D loop intermediate mediated by AtRAD51 and AtDMC1 and other proteins. If the second end is captured and the broken DNA strands are ligated, a dHJ is formed. This intermediate is resolved as CO^1^, sensitive to interference, or NCO upon appropriate resolution of the two HJs. On the other hand, this dHJ can be dissolved as a NCO. Alternatively, the D loop can be processed to generate a CO^2^ (insensitive to interference). When the D-loop is dissociated before the second end capture SDSA pathway occurs, the invading strand dissociates after DNA synthesis. This strand then re-anneals to the original parent, resulting in repair of the DSB and a heteroduplex DNA. This pathway is always resolved as a NCO.
(TIF)

S5 Fig DSBs are not produced in *Atspo11-1-5*. (A-C) Dual immunolocalization on *Atspo11-1-5* nuclei. (A) AtASY1 (green) and γH2AX (red). (B) AtASY1 (green) and AtRAD51 (red). (C) AtASY1 (green) and AtDMC1 (red). Bars = 5µm.
(TIF)

S6 Fig Chromosomal localization of the different NFTL alleles. (TIF)

S1 Table Comparisons of γH2AX, AtRAD51, AtDMC1, AtMSH4 and AtMLH1 foci between WT and *Atfas1-4*. (PDF)

S2 Table Tetrad analysis corresponding to the different NTFL alleles tested in *Atfas1-4*. All the analyzed plants were heterozygous for the fluorescent and non-fluorescent allele and homozygous for *Atfas1-4*.
(PDF)

S3 Table Comparisons of SIP between the different mutants analyzed. (PDF)

S4 Table Comparisons of SC lengths between the different mutants analyzed. (PDF)

S5 Table Genotyping primers. (PDF)

S6 Table qPCR primers. (PDF)
