# Supplementary figures and images for: Analysis of the Relationships between DNA Double-Strand Breaks, Synaptonemal Complex and Crossovers Using the Atfas1-4 Mutant
Source: PLoS Genet. 2015 Jul 6;11(7):e1005301. doi: 10.1371/journal.pgen.1005301 (PMC4492999; doi:10.1371/journal.pgen.1005301)

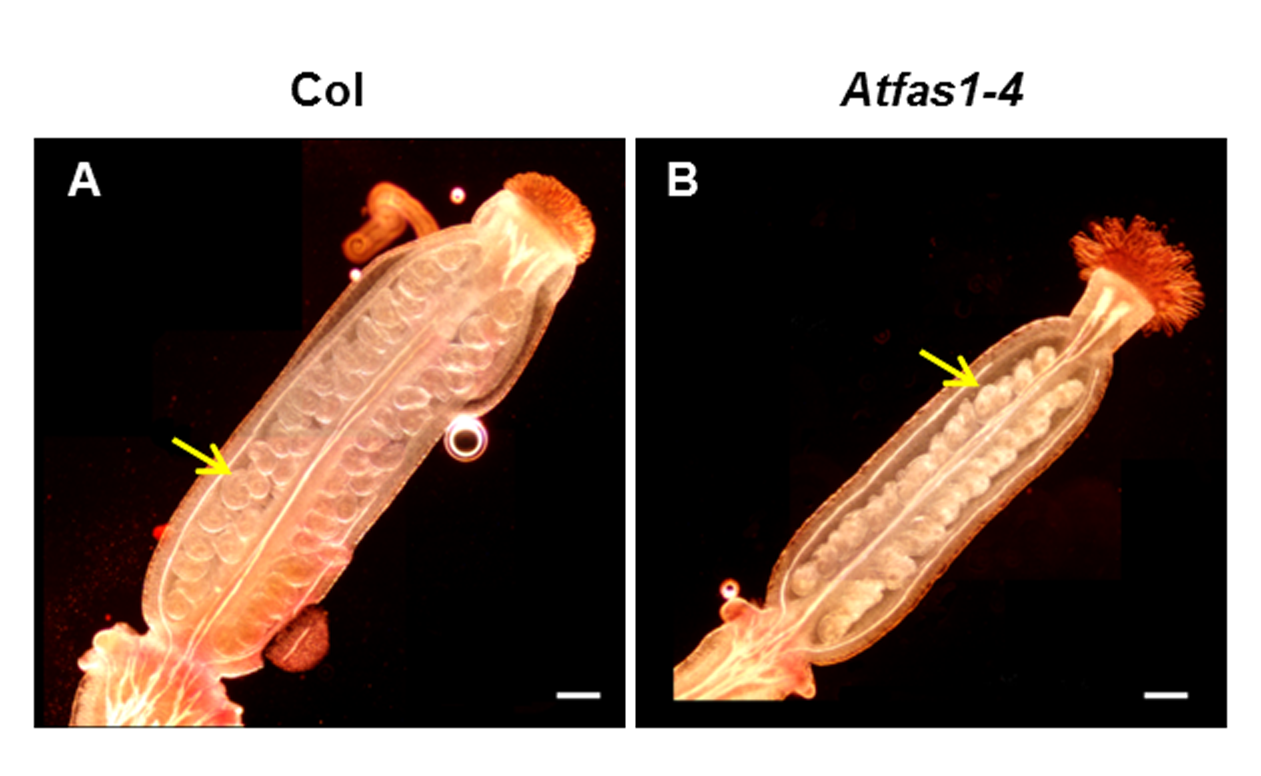

Supplement: S1 Fig — Squash procedure to visualize and estimate the number of ovules per gynoecium (A) on WT and (B) on Atfas1-4. (TIF) [file pgen.1005301.s001.tif]

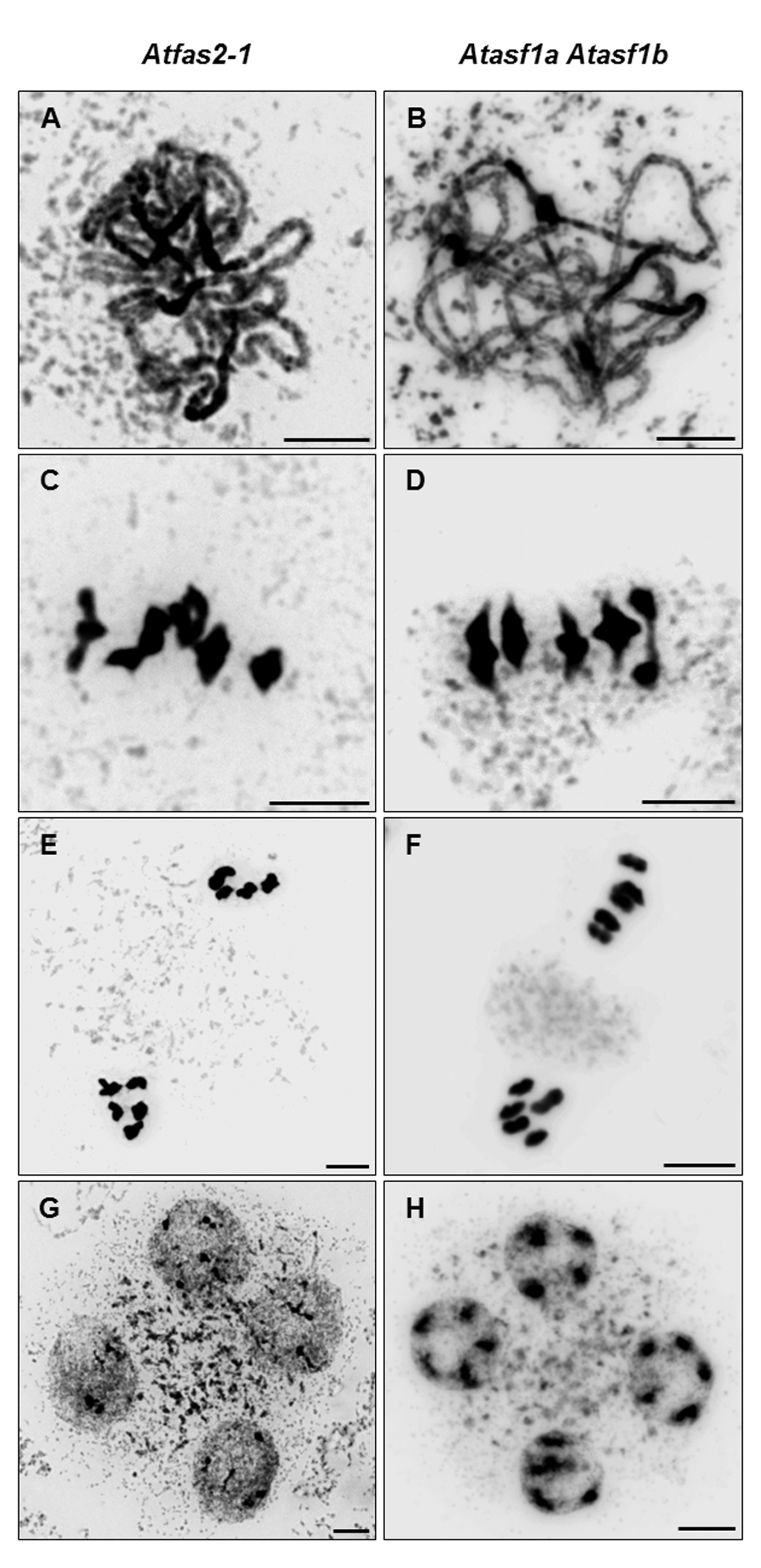

Supplement: S2 Fig — Chromosome spread preparations from (A, C, E, G) Atfas1-2 and (B, D, F, H) AtASF1a/AtASF1b RNAi PMCs. (A, B) Pachytene. (C, D) Metaphase I: four ring bivalents and one rod bivalent on each. (E, F) Prophase II. (G, H) Tetrad. Bars = 5µm. (TIF) [file pgen.1005301.s002.tif]

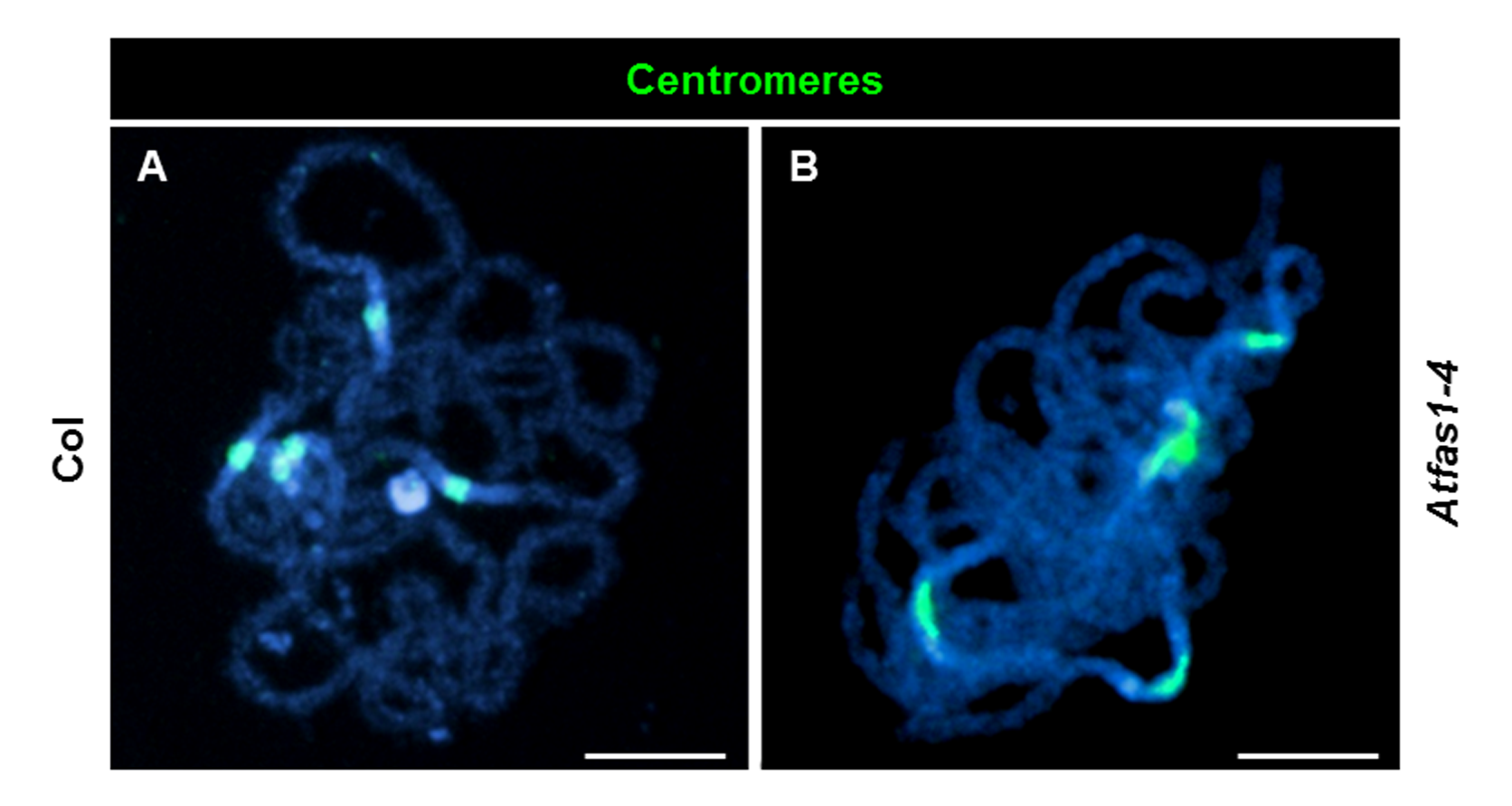

Supplement: S3 Fig — FISH using the centromeric probe pAL1 (180 bp) (A) on WT and (B) on Atfas1-4 at pachytene. In the mutant the centromeric FISH signal is weaker, less defined and more extended than in WT. (TIF) [file pgen.1005301.s003.tif]

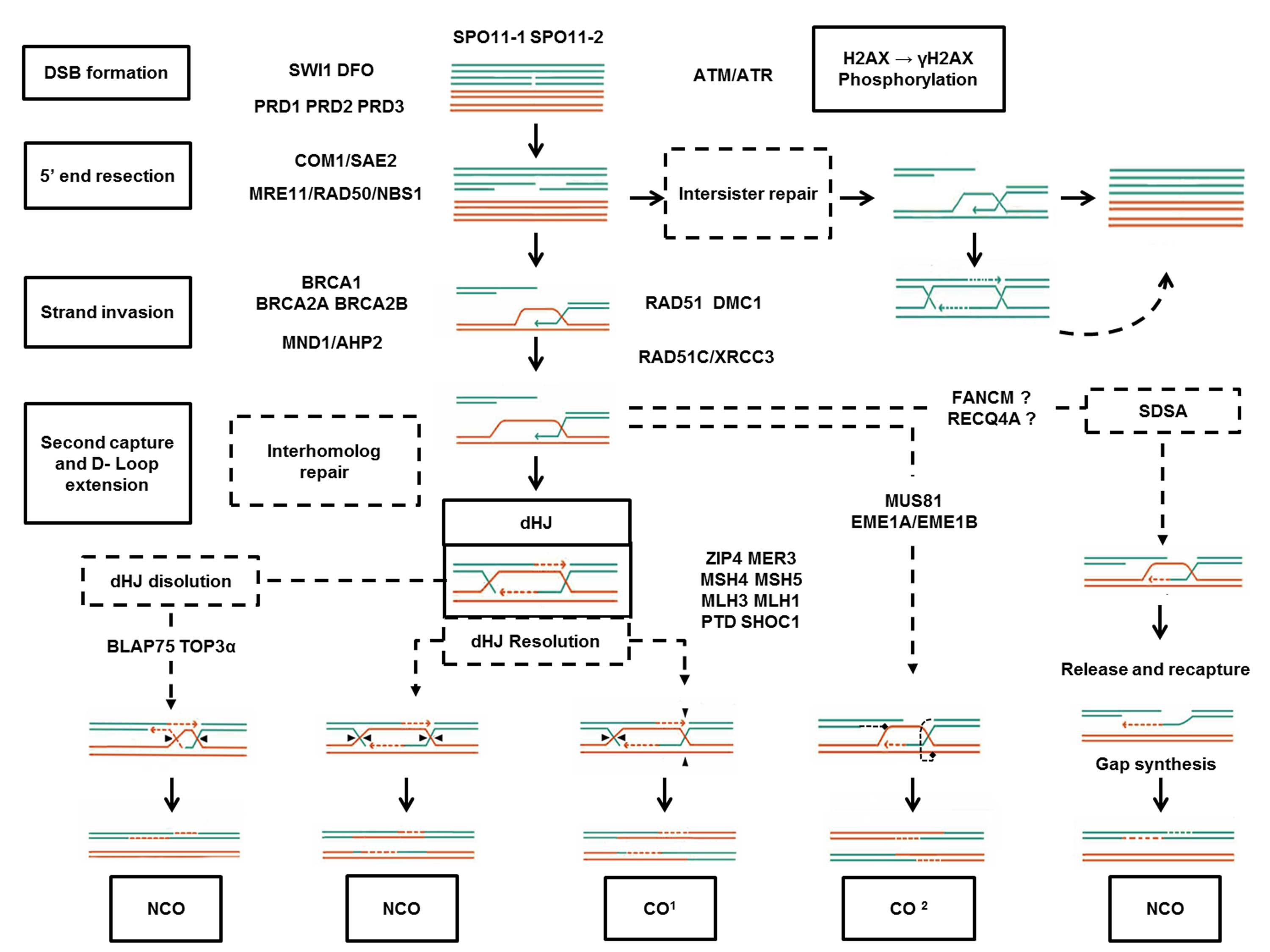

Supplement: S4 Fig — Single strands of DNA are shown as either blue (parent 1) or red (parent 2) rods. AtSPO11 helped by other proteins initiates programmed DSBs. H2AX phosphorylation occurs at the break zone and DSBs are resected 5’ to 3’ to produce single ssDNA tails by the MRN complex and COM1. One of these ends invades the homologous duplex DNA, giving raise a D loop intermediate mediated by AtRAD51 and AtDMC1 and other proteins. If the second end is captured and the broken DNA strands are ligated, a dHJ is formed. This intermediate is resolved as CO1, sensitive to interference, or NCO upon appropriate resolution of the two HJs. On the other hand, this dHJ can be dissolved as a NCO. Alternatively, the D loop can be processed to generate a CO2 (insensitive to interference). When the D-loop is dissociated before the second end capture SDSA pathway occurs, the invading strand dissociates after DNA synthesis. This strand then re-anneals to the original parent, resulting in repair of the DSB and a heteroduplex DNA. This pathway is always resolved as a NCO. (TIF) [file pgen.1005301.s004.tif]

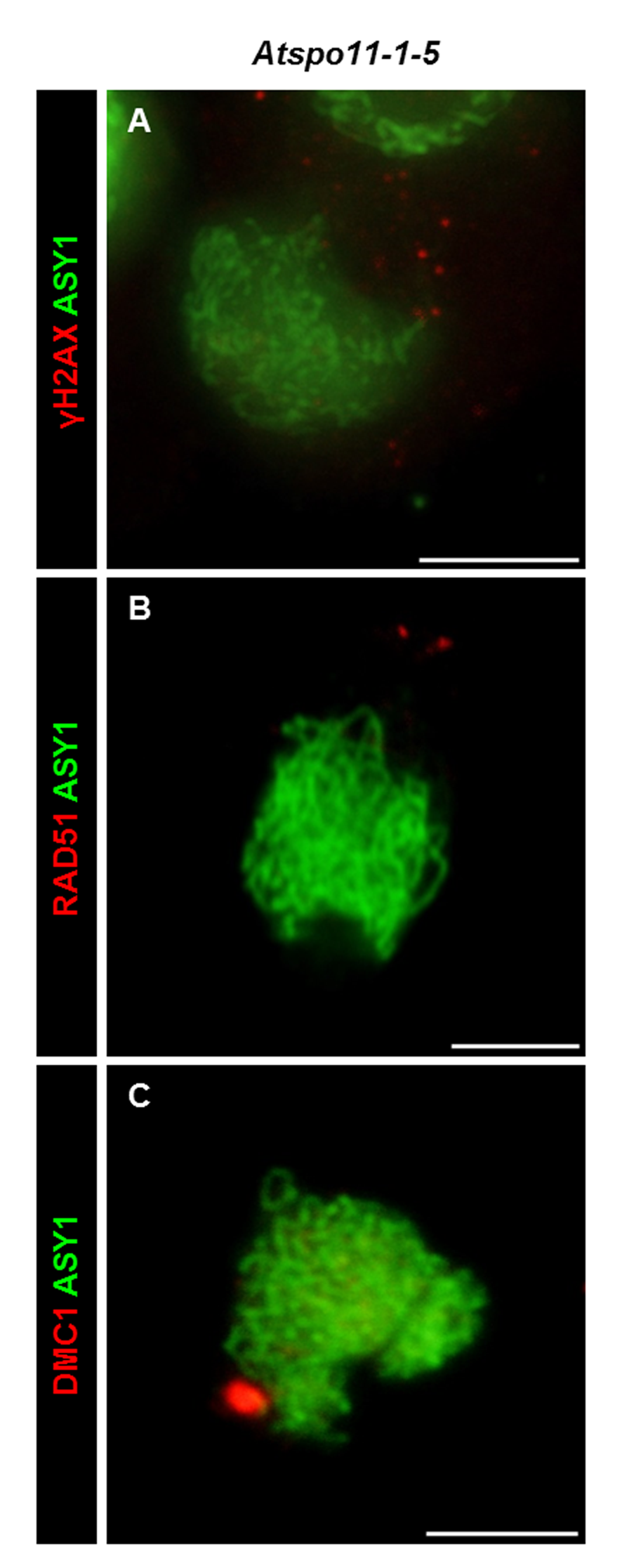

Supplement: S5 Fig — (A-C) Dual immunolocalization on Atspo11-1-5 nuclei. (A) AtASY1 (green) and γH2AX (red). (B) AtASY1 (green) and AtRAD51 (red). (C) AtASY1 (green) and AtDMC1 (red). Bars = 5µm. (TIF) [file pgen.1005301.s005.tif]

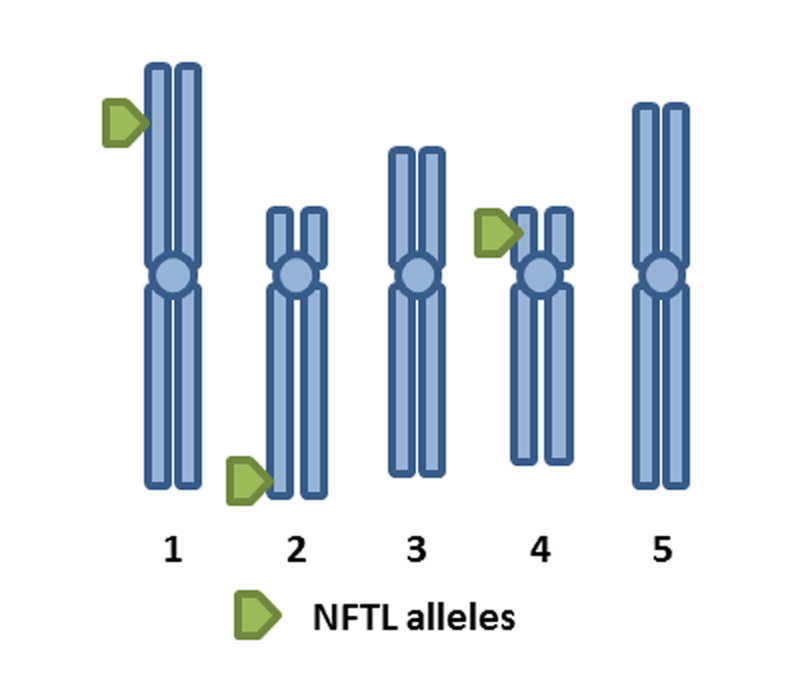

Supplement: S6 Fig — (TIF) [file pgen.1005301.s006.tif]
