# Supplementary material for: Analysis of the Relationships between DNA Double-Strand Breaks, Synaptonemal Complex and Crossovers Using the Atfas1-4 Mutant
Source: PLoS Genet. 2015 Jul 6;11(7):e1005301. doi: 10.1371/journal.pgen.1005301 (PMC4492999; doi:10.1371/journal.pgen.1005301)
Supplement: S1 Table — (PDF) [file pgen.1005301.s007.pdf]

S1 Table. Comparisons of γH2AX, AtRAD51, AtDMC1, AtMSH4, AtMLH1 and AtMUS81 foci between WT and *Atfas1-4*.

	**Col**	***Atfas1-4***
**γH2AX**	114.17 ± 5.29	180.05 ± 5.7
	(92-149)	(100-231)
	n = 20	n = 21
**AtRAD51**	120.55 ± 2.44	175.07 ± 6.42
	(94-155)	(121-225)
	n = 38	n = 28
**AtDMC1**	134.39 ± 3.82	179.50 ± 6.49
	(96-159)	(143 - 247)
	n = 25	n = 20
**AtMSH4**	123.60 ± 3.49	117.93 ± 5.57
	(96-159)	(54-124)
	n = 25	n = 20
**AtMLH1**	8.77 ± 0.42	9.06 ± 0.49
	(6-14)	(6-13)
	n = 20	n = 20
**AtMUS81**	1.86 ± 0.22	1.73 ± 0.18
	(1-3)	(1-3)
	n = 15	n = 14
The values in parentheses represent the range of variation. n, number of cells analyzed.		
