# Supplementary material for: Analysis of the Relationships between DNA Double-Strand Breaks, Synaptonemal Complex and Crossovers Using the Atfas1-4 Mutant
Source: PLoS Genet. 2015 Jul 6;11(7):e1005301. doi: 10.1371/journal.pgen.1005301 (PMC4492999; doi:10.1371/journal.pgen.1005301)
Supplement: S2 Table — All the analyzed plants were heterozygous for the fluorescent and non-fluorescent allele and homozygous for Atfas1-4. (PDF) [file pgen.1005301.s008.pdf]

S2 Table. Tetrad analysis corresponding to the different NFTL alleles tested in *Atfas1-4*. All the analyzed plants were heterozygous for the fluorescent and non-fluorescent allele and homozygous for *Atfas1-4*.

Chromosomal localization of the NFTL allele	NFTL allele	Plant	2:2 Tetrads	3:1 Tetrads	2:2 Total	3:1 Total	Total
1	NFTL 567	1	6031	5	11769	12	11781
		2	1703	2			
		3	1987	2			
		4	922	1			
		5	1126	2			
2	NFTL 3411	1	1720	3	7839	7	7846
		2	1207	1			
		3	1721	1			
		4	1357	1			
		5	1834	1			
4	NFTL 424	1	1325	1	7309	6	7315
		2	1672	1			
		3	1450	1			
		4	1592	1			
		5	1270	2			
