# Supplementary material for: Analysis of the Relationships between DNA Double-Strand Breaks, Synaptonemal Complex and Crossovers Using the Atfas1-4 Mutant
Source: PLoS Genet. 2015 Jul 6;11(7):e1005301. doi: 10.1371/journal.pgen.1005301 (PMC4492999; doi:10.1371/journal.pgen.1005301)
Supplement: S3 Table — (PDF) [file pgen.1005301.s009.pdf]

S3 Table. Comparisons of SIPs between the different mutants analyzed.

	Col	*Atfas1-4*	*Atspo11-1-5*	*Atfas1-4 Atspo11-1-5*	*Atdmc1-2*	*Atfas1-4 Atdmc1-2*	*Atrad51-3*	*Atfas1-4 Atrad51-3*	*Atrad51-2*
Atfas1-4	-								
Atspo11-1-5	***	***							
Atfas1-4 Atspo11-1-5	-	-	***						
Atdmc1-2	*	***	***	***					
Atfas1-4 Atdmc1-2	-	-	***	-	***				
Atrad51-3	-	-	***	**	***	**			
Atfas1-4 Atrad51-3	*	*	***	-	***	-	***		
Atrad51-2	-	-	***	-	***	-	-	*	
Atfas1-4 Atrad51-2	***	***	***	-	***	**	***	*	***

Wilcoxon Mann-Whitney test. ***$P < 0.001$; **$P < 0.01$; *$P < 0.05$; - Not significant
