# Supplementary material for: Analysis of the Relationships between DNA Double-Strand Breaks, Synaptonemal Complex and Crossovers Using the Atfas1-4 Mutant
Source: PLoS Genet. 2015 Jul 6;11(7):e1005301. doi: 10.1371/journal.pgen.1005301 (PMC4492999; doi:10.1371/journal.pgen.1005301)
Supplement: S4 Table — (PDF) [file pgen.1005301.s010.pdf]

S4 Table. Comparisons of SC lengths between the different mutants analyzed.

	Col	*Atfas1-4*	*Atspo11-1-5*	*Atfas1-4 Atspo11-1-5*	*Atdmc1-2*	*Atfas1-4 Atdmc1-2*	*Atrad51-3*	*Atfas1-4 Atrad51-3*	*Atrad51-2*
Atfas1-4	-								
Atspo11-1-5	***	**							
Atfas1-4 Atspo11-1-5	***	**	***						
Atdmc1-2	***	***	***	***					
Atfas1-4 Atdmc1-2	***	**	***	**	-				
Atrad51-3	***	***	***	**	***	-			
Atfas1-4 Atrad51-3	***	**	***	**	***	***	***		
Atrad51-2	***	**	***	-	***	**	**	**	
Atfas1-4 Atrad51-2	***	**	***	***	***	***	***	***	***

Wilcoxon Mann-Whitney test. ***P < 0.001; **P < 0.01; *P < 0.05; - Not significant
