# Supplementary material for: Analysis of the Relationships between DNA Double-Strand Breaks, Synaptonemal Complex and Crossovers Using the Atfas1-4 Mutant
Source: PLoS Genet. 2015 Jul 6;11(7):e1005301. doi: 10.1371/journal.pgen.1005301 (PMC4492999; doi:10.1371/journal.pgen.1005301)
Supplement: S5 Table — (PDF) [file pgen.1005301.s011.pdf]

S5 Table. Genotyping primers.

Primer	Sequence 5'-3'	Collection
**T-DNA specific primers**		
**LBa1**	TGGTTCACGTAGTGGGCCATCG	SALK
**LB2-SAIL**	GCTTCCTATTATATCTTCCCAAATTACCAATACA	SAIL
**LB2**	TGCCAGGTGCCCACGGAATAG	JP (Spanish collection)
**FTL-L1**	CAATTCGGCGTTAATTCAGTAC	NFTL
**FTL-L2**	CTATGTTACTAGATCGACCGG	NFTL
**Line specific primers**		
Atfas1-4	AAGGAACAAGCCGAGCTAAAG	SAIL
	CAAGTTGTAAAGCCACGTCGT	
Atspo11-1-5	AAATGCCACAATGGAGGTATG	SALK
	CAGTTTCTCTCAGGCATTTCG	
Atrad51-3	TCTCAAGAACTTTGCAAGATGC	SALK
	ATGCCAAGGTTGACAAGATTG	
Atrad51-2	AAGCATCACCATCTCCAATG	JP (Spanish collection)
	GCCCCAGAAAAAATCTTCCAG	
Atdmc1-2	GACTCATTGTTGCTTGATCCC	SAIL
	TCCACTCGGAATAAAGCAATG	
**NFTL 567-GC1**	TGGTCGGCCCTAAATGTTTG	NFTL
	ACCGACACAAGAATCTGTGGAACC	
**NFTL 3411-GC1**	CAAACCGATTTGCTGTGAACTC	NFTL
	GGACGTGGTATGTATTAGTTAGCC	
**NFTL 424-GC1**	CGGACTCTGTCTTCTCCACAAA	NFTL
	CTCAGTTAGTTGACTTTACAGC	
