# Supplementary material for: Analysis of the Relationships between DNA Double-Strand Breaks, Synaptonemal Complex and Crossovers Using the Atfas1-4 Mutant
Source: PLoS Genet. 2015 Jul 6;11(7):e1005301. doi: 10.1371/journal.pgen.1005301 (PMC4492999; doi:10.1371/journal.pgen.1005301)
Supplement: S6 Table — (PDF) [file pgen.1005301.s012.pdf]

S6 Table. qPCR primers.

Gene	AGI code	Sequence 5´- 3´	UPL probe	Length (bp)
AtSWI1	At5g51330	TTATTGGAGAAACCTTGCGTAAA	69	60
		CCACTGTTTTCTTAAGCCATCC		
AtSPO11-1	At3g13170	TTCCCAAACAGTGTCTTTTGC	143	113
		TTCAAGTTCCAACCTCCATTG		
AtSPO11-2	At1g63990	TTGGTATTATGGCTTCAAGCAG	11	76
		TCTACAGCTTCCTTACCTGGTTCT		
AtPRD1	At4g14180	GCTCTAGCATTCACGTTACTTCTCT	78	93
		GCCAAAGAAAGTTTGCCAAT		
AtMRE11	At5g54260	GTTTCCGCCAGTCTCAAAGA	8	74
		TTCTCCAATGGTGGAAGCA		
AtRAD50	At2g31970	GCAGTGCAGGTCAAAAGGTT	136	118
		GGCCCATCCAGGTTTGTAG		
AtNBS1	At3g02680	TGCGAAGGATCCATACAAAGA	38	99
		AAGTCCTCTGCAATGGCTTC		
AtCOM1	At3g52115	CAGCATGAGAAATCAGCAATCT	68	93
		CGAGCCAGTACCAATTCTCAC		
AtATM	At3g48190	AGGGTGGTGAGATGAGAAGC	98	67
		TCTGTGTCAATTGCGTCTTGT		
AtATR	At5g40820	TTCAGCGCCCAAAGAAGA	3	67
		GGCTTGCAGAGGAATGGATA		
AtBRCA1	At4g21070	CCAAGAAATTGGTCTTATCTTGC	100	73
		AGTTCCGCAAATTCTGCAAT		
AtBRCA2B	At5g01630	CACCTTAAAACCCGCAGTG	140	117
		AGGTGATTTACAAGCACCGATT		
AtRAD51C	At2g45280	TCAACTAGCGCTTGCTTTAGG	54	65
		AATACAGAATGACTCGGTTGGTG		
AtRAD51	At5g20850	CATGCCACCACAACAAGG	91	78
		ACATGGCGAGCTTATCACTTTAC		
AtDMC1	At3g22880	TCAACGTTGCTGTCTACATGACT	31	90
		GACCACCTGCTGGCTTTTT		
AtMND1	At4g29170	TGCGAAAGACAAGATTGGAA	33	88
		AAGTTTCTGGCGAACACTCC		
AtAHP2	At1g13330	GAACATTACGGTTGCGTTGTT	53	60
		GGCTGCGTCAACCAATAGTC		
AtMSH4	At4g17380	CAAGAATGGGGACAATGGAT	151	82
		TGCATTATGAAAGCGGTCTCT		
AtMLH3	At4g35520	GACTGAAGCAGACCTCACTTTG	47	76
		GCCTTCAAATCGACAAGAGG		
AtMUS81	At4g30870	GATATGTACCCAACGCTTTTGTC	29	73
		CTTCTTGCGCCGAGACAT		
AtBLAP75	At5g63540	TTCCAGTTGTTTCTCGCTTGA	157	63
		TTGCGACGATCTGTTCATTT		
AtTOP3α	At5g63920	GGCTTTGATGTTAATCACTTAGGTT	76	76
		TTCCACAGATGTCTATCAACTGTTT		
AtSMC6A	At5g07660	TGCCTCAAGATGCAACAAAC	150	76
		AAAGTCGAGAAAGACCGTTCC		
AtSMC6B	At5g61460	TCGCACGAGAGGATAAAGAAA	68	106
		TGACTCAAAGCCGAGGATG		
